# Tanshinone I attenuates fibrosis in fibrotic kidneys through down-regulation of inhibin beta-A

**DOI:** 10.1186/s12906-022-03592-3

**Published:** 2022-04-19

**Authors:** Ming Wu, Feng Yang, Di Huang, Chaoyang Ye

**Affiliations:** 1 Department of Nephrology, Shuguang Hospital Affiliated to Shanghai University of Traditional Chinese Medicine, No.528 Zhangheng Road, Pudong District, Shanghai, 201203 PR China; 2 TCM Institute of Kidney Disease of Shanghai University of Traditional Chinese Medicine, Shanghai, China; 3 Key Laboratory of Liver and Kidney Diseases, Ministry of Education, Shanghai Key Laboratory of Traditional Chinese Clinical Medicine, Shanghai, China

**Keywords:** Tanshinone I, Renal fibrosis, INHBA, CKD

## Abstract

**Background:**

Tanshinone I (Tan-I), an ingredient of Salvia miltiorrhiza, displays protective effects in several disease models. We aim to study the effect of Tan-I on renal fibrosis and explore its underlining mechanism.

**Methods:**

Rat renal fibroblasts (NRK-49F) were used as an *in vitro* model to study the effect of Tan-I. Mouse renal fibrosis model was induced by unilateral ureteral obstruction (UUO) or peritoneally injection of aristolochic acid I (AAI).

**Results:**

We found that Tan-I dose-dependently inhibited the expression of pro-fibrotic markers in rat renal fibroblasts. Masson staining and Western blotting analysis showed that Tan-I treatment attenuated renal fibrosis in UUO or AAI induced fibrotic kidneys. RNA sequencing analysis identified inhibin beta-A (INHBA), a ligand of TGF-β superfamily, as a downstream target of Tan-I in fibrotic kidneys, which were further verified by qPCR. Western blotting analysis showed that INHBA is up-regulated in UUO or AAI induced fibrotic kidneys and Tan-I reduced the expression of INHBA in fibrotic kidneys. Inhibition of INHBA by Tan-I was further confirmed in rat fibroblasts. Moreover, knockdown of INHBA reduced the expression of pro-fibrotic markers and abolished the ani-fibrotic effect of Tan-I in rat renal fibroblasts.

**Conclusions:**

We conclude that Tan-I attenuates fibrosis in fibrotic kidneys through inhibition of INHBA.

**Supplementary Information:**

The online version contains supplementary material available at 10.1186/s12906-022-03592-3.

## Background

Chronic kidney disease (CKD) is a common human disease and epidemiological studies show that the prevalence of CKD in adults is as high as more than 10%, resulting in a huge social and economic burden [1]. Renal interstitial fibrosis is the common pathway and main pathological basis for the progression of various CKDs to end-stage renal disease [2]. Pathological characterization of renal interstitial fibrosis is the excessive deposition of extracellular matrix (ECM) in the kidney, which is due to the persistence of pathogenic causes and the imbalance of damage and repair in the inflammatory process leading to secretion of a variety of pro-fibrotic factors such as transforming growth factor beta (TGF-β) [2, 3].

TGF-β is one of the most important factors leading to the development of renal fibrosis, and Smad3 is an important downstream molecule of TGF-β signaling pathway [2, 4, 5]. Inhibition or knockout of Smad3 reduces renal fibrosis in diabetic nephropathy and ureteral obstruction nephropathy [6, 7]. Through activation of Smad pathway, TGF-β promotes the occurrence of epithelial mesenchymal transition (EMT), which is featured by expression of mesenchymal cell markers such as N-cadherin, fibronectin (FN) and α smooth muscle actin (α-SMA) [4]. In fibrotic kidneys, only partial EMT occurs which means renal tubular epithelial cells do not fully transdifferentiate into interstitial fibroblasts, but still integrate into renal tubules. Partial transdifferentiated renal tubular cells produce pro-fibrotic factors and thus promote renal fibrosis progression [4, 8]. Snail is an important transcriptional factor triggering partial EMT and promotes renal fibrosis [8].

Tanshinone I (Tan-I) is a fat-soluble compound extracted from Salvia miltiorrhiza [9]. The effect of Tan-I was extensively studied in the field of cancer biology [10–12]. Tan-I inhibits the growth and metastasis of osteosarcoma through inhibition of JAK/STAT3 signaling pathway [12]. Tan-I exerts inhibitory effect on tumor growth through inducing apoptosis and enhancing autophagy of ovarian cancer cells [11]. It has also been reported that Tan-I enhances the radio-sensitivity of lung cancer cells [10]. The protective effect of Tan-I in other diseases has also been shown. Tan-I induces apoptosis of activated hepatic stellate cells suggesting that Tan-I is a potential novel agent for treatment of hepatic fibrosis [13]. It has been shown that Tan-I protects against lung inflammation by activating the Nrf2-dependent antioxidant response [14]. Studies of Tan-I in renal disease is limited. The renal protective effect of Tan-I has been reported in aristolochic acid-induced acute renal injury in rats through induction of hepatic cyp1a [15]. Whether Tan-I is anti-fibrotic in renal disease is yet to be determined.

Inhibin beta-A (INHBA), a ligand of TGF-β superfamily, acts as a subunit of inhibin A (activin A) to exert a variety of biological functions and can promote the differentiation of embryonic cells during early embryogenesis [16, 17]. Silencing of INHBA inactivates the TGF-β signaling pathway [18]. INHBA can also promote EMT by activating the TGF-β signaling pathway [19]. The role of INHBA in renal fibrosis is currently unclear.

The aim of current study is to investigate the effect of Tan-I on renal fibrosis and its downstream mechanisms.

## Methods

### Animal studies

Male C57 mice (C57bl/6j background, SPF grade, 20–25 g) were bought from B&K Universal Group Limited (Shanghai, China) and were housed in the animal facility of Shanghai University of Traditional Chinese Medicine according to local regulations and guidelines. The animal study was performed in accordance with ARRIVE guidelines. Animal experiments were approved by the ethic committee of Shanghai University of Traditional Chinese Medicine (PZSHUTCM18111601).

Mouse were anesthetized with 0.8% sodium pentobarbital (50 mg/kg body weight, i.p.) before UUO operation. UUO model was established by ligation of the left ureter as described previously [20, 21]. Mice were randomly divided into four groups: 1) Sham/DMSO (*n* = 9), 2) Sham/Tan-I (*n* = 8), 3) UUO/DMSO (*n* = 8), and 4) UUO/Tan-I (*n* = 11) group. Sham or UUO mice were treated with 20 μl DMSO or equivalent volume of Tan-I (50 mg/kg, Topscience, T2907, Shanghai, China) per day by intraperitoneal (i.p.) injection for 14 days starting from day0. Mice were anesthetized with sodium pentobarbital (50 mg/kg body weight, i.p.) and then executed at day14. The dose of Tan-I was taken based on two previous animal studies [15, 22].

To induce aristolochic acid nephropathy (AAN) in mice, aristolochic acid I (AAI, A9451, Sigma; 5 mg/kg body weight) was peritoneally injected two times at day 0 and day7. Mice were randomly divided into three groups: 1) Normal saline (NS)/DMSO (*n* = 8), 2) AAI/DMSO (*n* = 8), 3) AAI/Tan-I (*n* = 8). Mice were treated with 20 μl DMSO or Tan-I daily start from day 14 for two weeks at day 28, and were anesthetized with sodium pentobarbital (50 mg/kg body weight, i.p.) and then executed. Finally, mice were euthanized by cervical dislocation under anesthesia. Kidney tissues were collected.

### Cell culture

Normal rat kidney 49F (NRK-49F) cells, a rat kidney interstitial fibroblast cell line, were purchased from national infrastructure of cell line resource, Chinese academy of medical sciences. NRK-49F cells were cultured in DMEM/F12 medium containing 10% fetal bovine serum and 0.5% penicillin and streptomycin in an atmosphere of 5% CO2 and 95% air at 37 °C according to previous studies [20, 21]. To study the anti-fibrotic effect of Tan I, NRK-49F cells were seeded in 6-well plates to 40–50% confluence and were starved with DMEM/F12 medium containing 0.5% fetal bovine serum overnight before the experiment. The next day, cell were refreshed with 0.5%medium and then were exposed to different concentrations of Tan-I (0.5 μM, 5 μM and 50 μM) (Topscience, T2907, Shanghai, China) for 24 h. Protein was extracted from cell lystates for further analysis.

Nonsense control (NC) or rat INHBA siRNA were transfected by Lipofectamine 2000 (11,668–027; Invitrogen) in NRK-49F cells using DMEM/F12 medium containing 10% fetal bovine serum according to the manufacturer’s instruction. Protein was extracted from cell lysates at 24 h after transfection. In another experiment, cells were exposed to vehicle or 50 μM Tan-I for another 24 h. The NC siRNA sequences were as follows: forward, 5′-UUCUCCGAACGUGUCACGUTT-3′; and reverse, 5′-ACGUGACACGUUCGGAGAATT-3′. The rat INHBA siRNA sequences were as follows: forward, 5′-GGGAUGAGGCCGAGGAAAUTT-3′; and reverse, 5′-AUUUCCUCGGCCUCAUCCCTT-3′.

### Quantitative real-time PCR and RNA sequence (RNA-seq)

Total RNA was extracted using Trizol (R401-01, Vazyme, Nanjing, China) from kidney samples according to the manufacture’s instruction, which was reverse transcribed to cDNA by Takara PrimeScript RT reagent kit (RR0036A, Kyoto, Japan). The primer sequences for quantitative Polymerase Chain Reaction (qPCR) were listed as follows: mouse INHBA forward, 5'-ATCATCACCTTTGCCGAGTCA-3'; mouse INHBA reverse, 5'-TTCTGCACGCTCCACTACTGA-3'; mouse GAPDH forward, 5'-AGGTCGGTGTGAACGGATTTG-3'; mouse GAPDH reverse, 5'-TGTAGACCATGTAGTTGAGGTCA-3'.

For RNA-seq, kidney samples were prepared from UUO/DMSO and UUO/Tan-I groups with three biological replicates. Total RNA was extracted from the kidney samples using TRIzol® Reagent with removal of genomic DNA by DNase I (TaKara). RNA quality was determined by 2100 Bioanalyser (Agilent) and quantified using the ND-2000 (NanoDrop Technologies). Only qualified RNA samples (OD260/280 = 1.8 ~ 2.2, OD260/230 ≥ 2.0, RIN ≥ 6.5, 28S:18S ≥ 1.0, > 1 μg) were further used for Illumina sequencing. The RNA purification, reverse transcription, library construction, and sequencing were performed at Majorbio Bio-pharm Biotechnology Co., Ltd (Shanghai, China) using Illumina HiSeq X10 (Illumina, San Diego, CA) according to the manufacturer’s instructions. All identified sequences were mapped with Gene Ontology Terms (GO, http://geneontology.org/) to determine the functional and biological properties. To identify DEGs (differential expression genes) between two different samples, the expression level of each transcript was calculated according to the fragments per kilobase of exon per million mapped reads method. Gene abundances was quantified by RSEM (RNA-Seq by Expectation–Maximization). In addition, to identify which DEGs were significantly enriched in metabolic pathways functional-enrichment analyses including Kyoto encyclopedia of genes and genomes (KEGG) were performed at Bonferroni-corrected P value ≤ 0.05 compared with the whole-transcriptome background. The data from this study was deposited in NCBI Sequence Read Archive under accession SRA: SRP347578.

### Western blotting analysis

As described previously [20, 21], protein from cells or mouse kidneys was extracted using RIPA lysis buffer (Beyotime Biotech) and measured by the bicinchoninic acid assay before being dissolved in laemmli sample buffer. Samples were subjected to SDS-PAGE gels and were electro-transferred to a PVDF membrane (Merck, Darmstadt, Germany), which was incubated in the blocking buffer (5% nonfat milk, 20 mM Tris HCl, 150 mM NaCl, pH 8.0, 0.1% Tween 20) for 1 h at room temperature. After that membrane was incubated with antibodies for fibronectin (FN; 1:1000, ab23750; Abcam, Cambridge, MA, USA), phosphorylated Smad3(1:1000, ET1609-41, Hangzhou, China, HUABIO, ɑ smooth muscle actin (ɑ-SMA; 1:1000, ET1607-53 Hangzhou, China, HUABIO), SNAIL(A11794, 1:1000, Wuhan, China, Abclonal), inhibin β A(INHBA, 1:1000, ER1911-46, Hangzhou, China, HUABIO; or 1:1000, A5232, Wuhan, China, Abclonal), ɑ-tubulin (1:1000, AF0001; Beyotime Biotech), and GAPDH (1:1000, 60,004–1-lg; Proteintech, Wuhan, Hubei, China) overnight at 4 °C. Binding of the primary antibody was detected by an ECL method (BeyoECL Star, P0018A; Beyotime Biotech) using horseradish peroxidase–conjugated secondary antibodies (goat anti-rabbit IgG, 1:1000, A0208; Beyotime Biotech; or goat anti-mouse IgG, 1:1000, A0216; Beyotime Biotech). The quantification of protein expression was performed by using -Image J (National Institutes of Health). For each figure, all detected proteins including the housekeeper protein were derived from the same membrane.

### Masson’s trichrome staining and quantification

Following the protocols described in a previous study [20, 21], mouse kidneys were fixed in 4% paraformaldehyde and further embedded in paraffin. Four-μm thick sections of paraffin-embedded kidney tissue was stained with hematoxylin, and then with ponceau red liquid dye acid complex, which was followed by incubation with phosphomolybdic acid solution. Finally, the tissue was stained with aniline blue liquid and acetic acid. Images were obtained by a microscope (Nikon 80i, Tokyo, Japan). Quantification of collagen positive area was performed by using the ImageJ software. The color threshold (the Hue was set to “125–220”; the saturation was “0–255” and the brightness was “150–225”) was set up to measure the blue positive area of collagen fibers staining. The total area was measured under the threshold mode “0–205”. Ten fields of view at 200 × magnification were captured from each pathological section clockwise, which covered at least 70% of the sample. The blue positive area was divided by the total area for each field, and the average value was calculated for each section.

### Statistical analysis

Results were presented as mean ± standard deviation (SD). The data were processed by using IBM SPSS Stastic 22.0 and GraphPad Prism version 8.0 for Windows (GraphPad Software, San Diego, California, United States). The normal distribution of the data was analyzed by Shapiro–Wilk test. When data were normally distributed, they were compared by unpaired student t-test to calculate significance between two groups. Non-normally distributed data were compared using non-parametric tests. A p value of lower than 0.05 was considered statistically significant.

## Results

### Tan-I inhibits fibrotic changes of rat renal fibroblasts

**Fig. 1:**
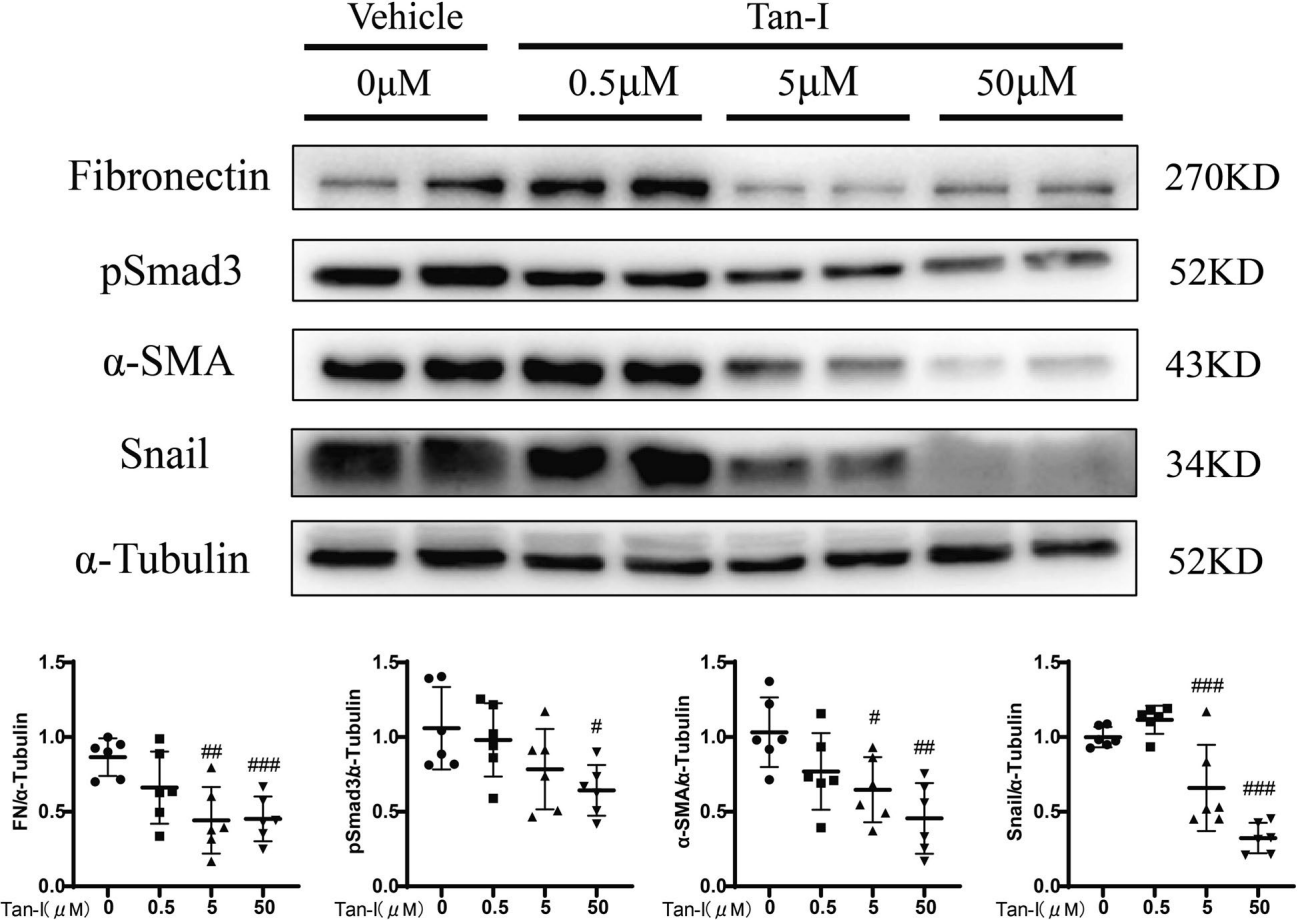
Tanshinone I (Tan-I) inhibits fibrotic changes of rat renal fibroblasts. Rat renal fibroblasts (NRK-49F) were starved for 24 h and followed by 24 h treatment with different concentration (0.5 μM, 5 μM, 50 μM) of Tan-I. The expression of fibronectin (FN),α-smooth muscle actin (α-SMA), Snail and phosphorylation of Smad3 (pSmad3) were analyzed by Western blotting and then quantified. Data represents mean ± SD. #*p* < 0.05 vs DMSO. ##*p* < 0.01 vs DMSO. *N* = 6 in each experimental group and one representative result of at least three independent experiments is shown. Full-length blots are presented in Supplementary Fig. 1

The anti-fibrotic effect of Tan-I was tested in rat renal fibroblasts (NRK-49F). Treatment with Tan-I for 24 h reduced the phosphorylation of Smad3 in a dose-dependent manner starting from 0.5 μM or 50 μM (Fig. 1). Moreover, the expression of EMT markers such as FN, Snail and α-SMA were reduced by Tan-I in NRK-49F cells (Fig. 1).

### Tan-I mitigates renal fibrosis in UUO mice

**Fig. 2:**
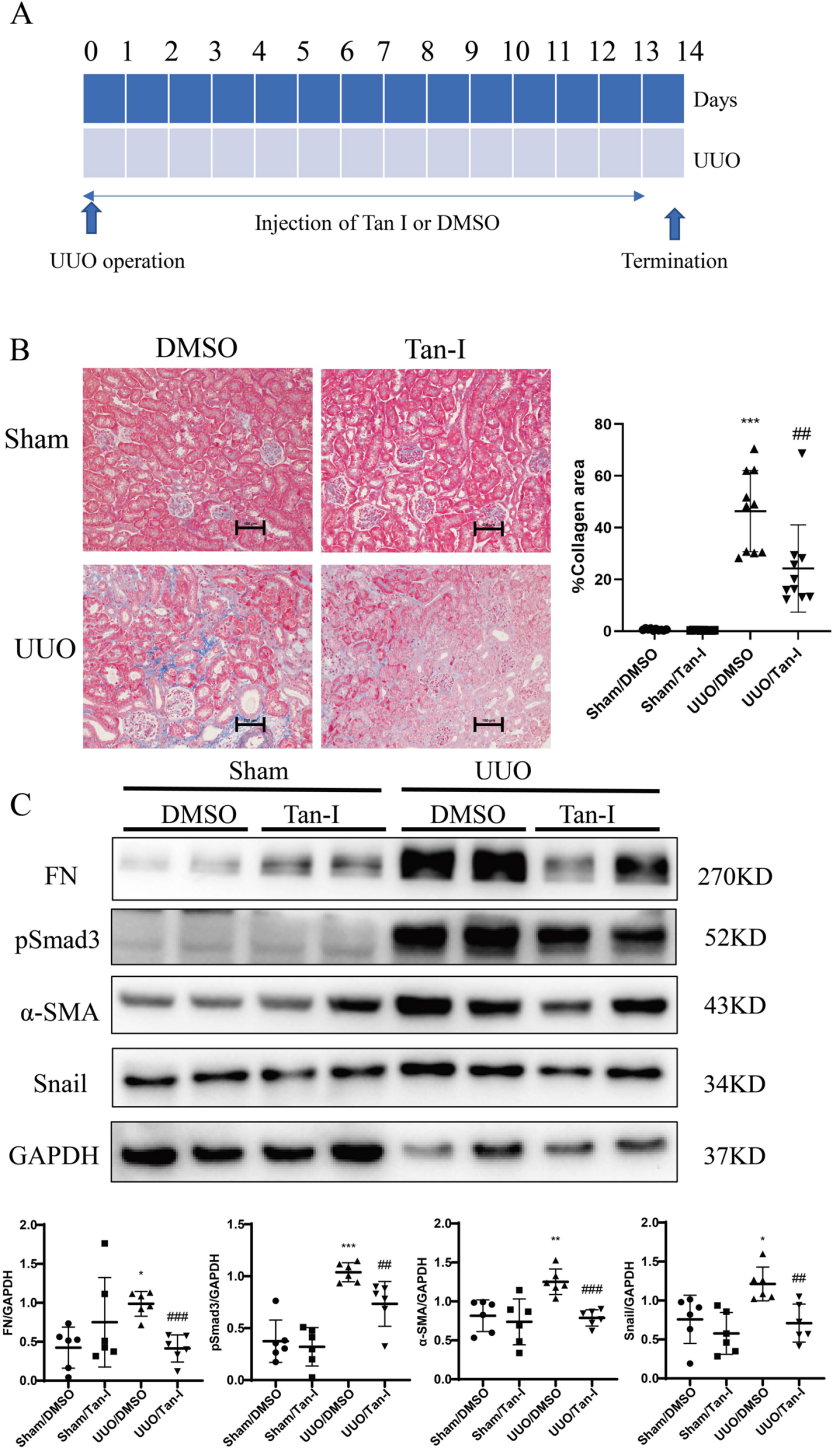
Tan-I mitigates renal fibrosis in UUO mice. **A** Study design. Wide type C57 mice received sham or UUO operation and followed by 14 days treatment with Tan-I. Mice were sacrificed at day 14. **B** Renal fibrosis was assessed by Masson’s trichrome staining and then quantified. Bars = 100 μm. **C** The expression of FN, α-SMA, Snail and pSmad3 were analyzed by Western blotting and then quantified. *N* = 8–11 in each experimental group and one representative of at least three independent experiments is shown. Data represents mean ± SD. *p < 0.05 vs Sham/DMSO. **p < 0.01 vs Sham/DMSO. ***p < 0.001 vs Sham/DMSO ^#^p < 0.05 vs UUO/DMSO. ^##^ *p* < 0.01 vs UUO/DMSO. ^###^ *p* < 0.001 vs UUO/DMSO. Full-length blots are presented in Supplementary Fig. 2

The anti-fibrotic effect of Tan-I was further tested in the mouse unilateral ureteral obstruction (UUO) model, a well-established model of progressive renal fibrosis (Fig. 2A) [23]. Masson trichrome staining revealed massive collagen deposition in UUO kidneys, which was significantly reduced by Tan-I (Fig. 2B). Western blotting further showed a significant up-regulation of FN, Snail, α-SMA and pSmad3 in UUO kidneys as compared with sham kidneys (Fig. 2C). Two weeks treatment with Tan-I significantly reduced the expression of FN, Snail, α-SMA and pSmad3 in UUO kidneys (Fig. 2C).

### Tan-I ameliorates renal fibrosis in AAN mice

**Fig. 3:**
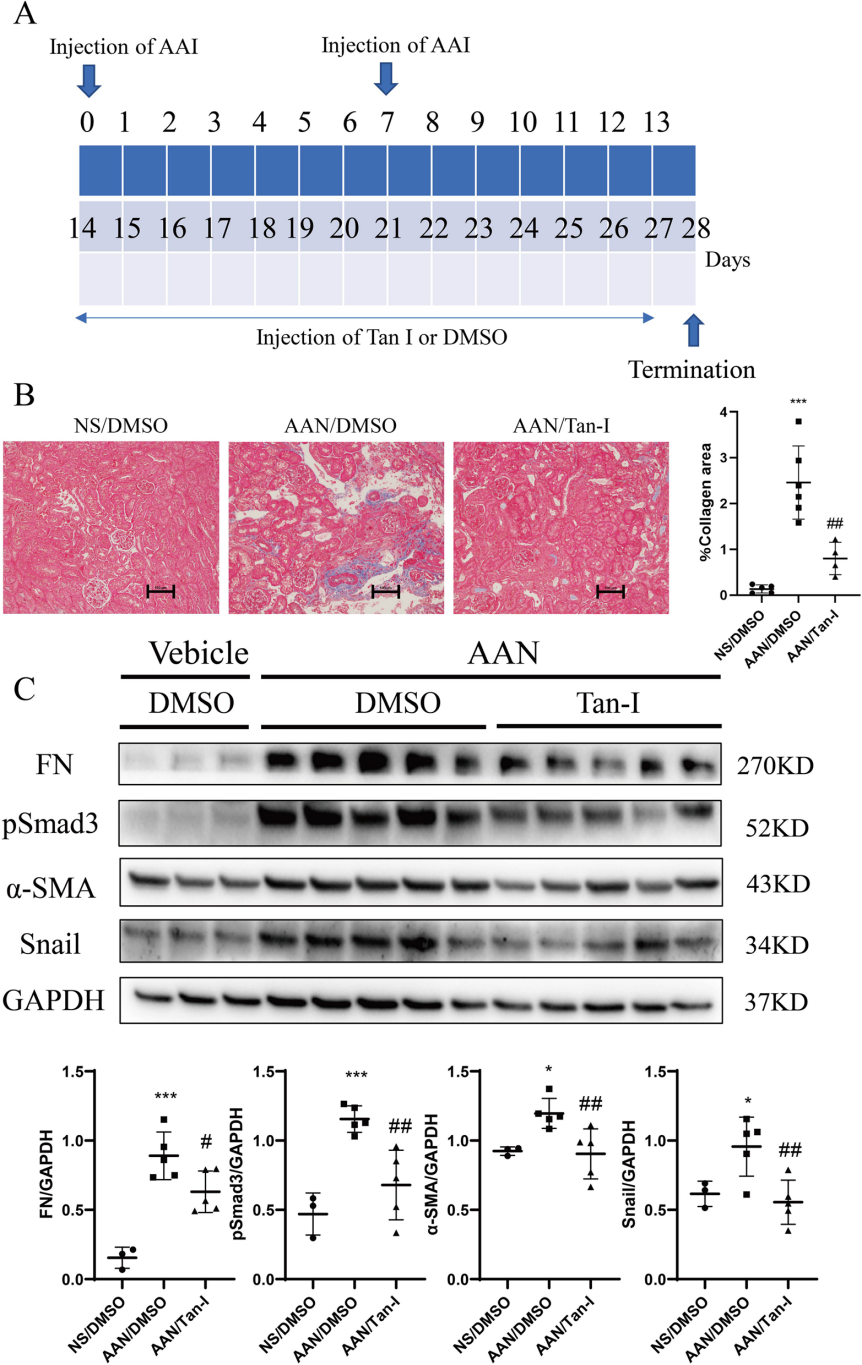
Tan-I ameliorates renal fibrosis in AAN mice. **A** Study design. Wide type C57 mice received two times peritoneally injection of aristolochic acid I (AAI) to induce aristolochic acid nephropathy (AAN). Mice were treated with Tan-I for two weeks from day14 and were sacrificed at day 28. **B** Renal fibrosis was assessed by Masson’s trichrome staining and then quantified. Bars = 100 μm. **C** The expression of FN, α-SMA, Snail1 and pSmad3 were analyzed by Western blotting and then quantified. *N* = 8 in each experimental group and one representative of at least three independent experiments is shown. Data represents mean ± SD. *p < 0.05 vs normal saline (NS)/DMSO. **p < 0.01 vs NS/DMSO. ***p < 0.001 vs NS/DMSO #*p* < 0.05 vs AAN/DMSO. ##*p* < 0.01 vs AAN/DMSO. ###*p* < 0.001 vs AAN/DMSO. Full-length blots are presented in Supplementary Fig. 3

Mouse aristolochic acid nephropathy (AAN) is a model to study chronic kidney disease (Fig. 3A) [24]. In our study, AAN was induced by two times peritoneally injection of aristolochic acid I (AAI). At 28 days after the first injection of AAI, a strong deposition of collagen was observed in AAN kidneys by Masson staining and two weeks treatment with Tan-I significantly reduced the Masson positive areas in AAN kidneys (Fig. 3B). Similarly, a significant up-regulation of FN, Snail, α-SMA and pSmad3 was observed in AAN kidneys which were reduced by Tan-I treatment (Fig. 3C).

### Tan-I reduces INHBA expression *in vitro* and *in vivo*

**Fig. 4:**
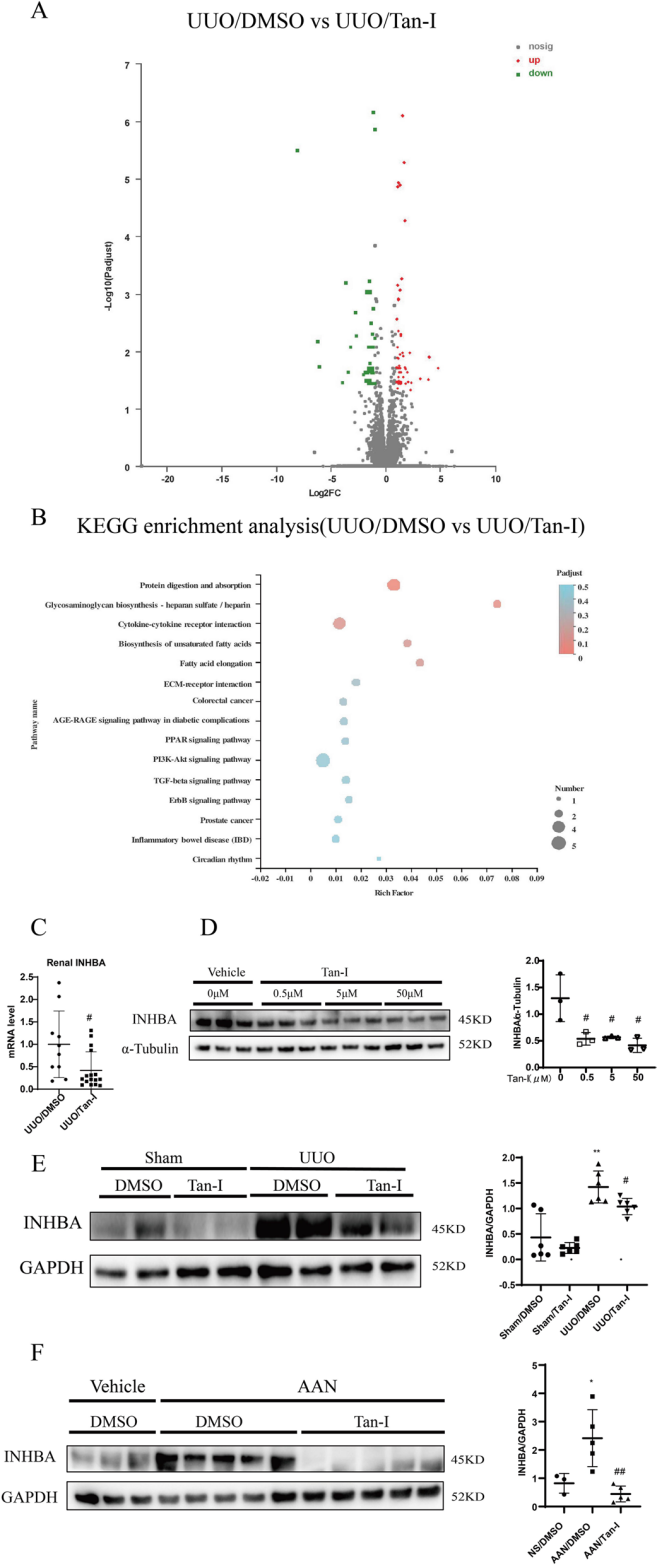
Tan-I reduces INHBA expression *in vitro* and *in vivo*. **A** The RNA from DMSO or Tan-I treated UUO kidneys was extracted and analyzed by RNA-Seq. **A** Volcano profiles of RNA sequence between the two groups was shown. **B** Kyoto encyclopedia of genes and genomes (KEGG) pathway analysis of differential expressed genes between two groups was performed [38–40]. **C** Quantitative PCR analysis of INHBA expression in UUO kidneys treated with DMSO or Tan-I. *N* = 8–11 in each experimental group and one representative of at least three independent experiments is shown. Data represents mean ± SD. ^#^ *p* < 0.05 vs UUO/DMSO. **D** NRK-49F cells were starved for 24 h and followed by 24 h treatment with different concentration (0.5 μM, 5 μM, 50 μM) of Tan-I. The expression of inhibin beta-A (INHBA) was analyzed by Western blotting and then quantified. Data represents mean ± SD. #*p* < 0.05 vs DMSO. ##*p* < 0.01 vs DMSO. *N* = 9 in each experimental group and one representative result of at least three independent experiments is shown. **E** The expression of INHBA in sham or UUO kidneys was analyzed by Western blotting and then quantified. Data represents mean ± SD. *p < 0.05 vs. Sham/DMSO. ##*p* < 0.01 vs UUO/DMSO. *N* = 8–11 in each experimental group and one representative of at least three independent experiments is shown. **F** The expression of INHBA in NS or AAN kidneys was analyzed by Western blotting and then quantified. Data represents mean ± SD. *p < 0.05 vs NS/DMSO. ##*p* < 0.01 vs AAN/DMSO. *N* = 8 in each experimental group and one representative of at least three independent experiments is shown. Full-length blots are presented in Supplementary Fig. 4

RNA sequence (RNA-seq) was performed to analyze the differential expressed genes between UUO/DMSO group and UUO/Tan-I group. Differential gene expression analysis revealed that 54 genes were up-regulated and 34 genes were down-regulated in Tan-I treated UUO kidneys (Fig. 4A), among which two genes (INHBA and BMP7) involved in TGF-β signaling pathway were identified by the Kyoto Encyclopedia of Genes and Genomes (KEGG) pathway analysis (Fig. 4B). RNA-seq analysis showed that the fold change of INHBA between UUO/DMSO group and UUO/Tan-I group was 0.297 (P value was 9.87669E-07). The fold change of BMP7 between UUO/DMSO group and UUO/Tan-I group was 2.455 (P value was 1.12E-04). However, we found no significant difference of BMP7 expression between UUO/DMSO and UUO/Tan-I groups by qPCR (data not shown). Down-regulation of INHBA gene in Tan-I treated UUO kidneys was confirmed by qPCR (Fig. 4C). Western blotting analysis showed that Tan-I dose-dependently the expression of INHBA in NRK-49F cells starting from 0.5 μM to 50 μM (Fig. 4D). INHBA was up-regulated in UUO kidneys as compared to sham kidneys and Tan-I significantly inhibited the expression of INHBA in UUO kidneys (Fig. 4E). Moreover, Tan-I blocked the increased expression of INHBA in AAN kidneys (Fig. 4F).

### Tan-I attenuates renal fibrosis through INHBA

**Fig. 5:**
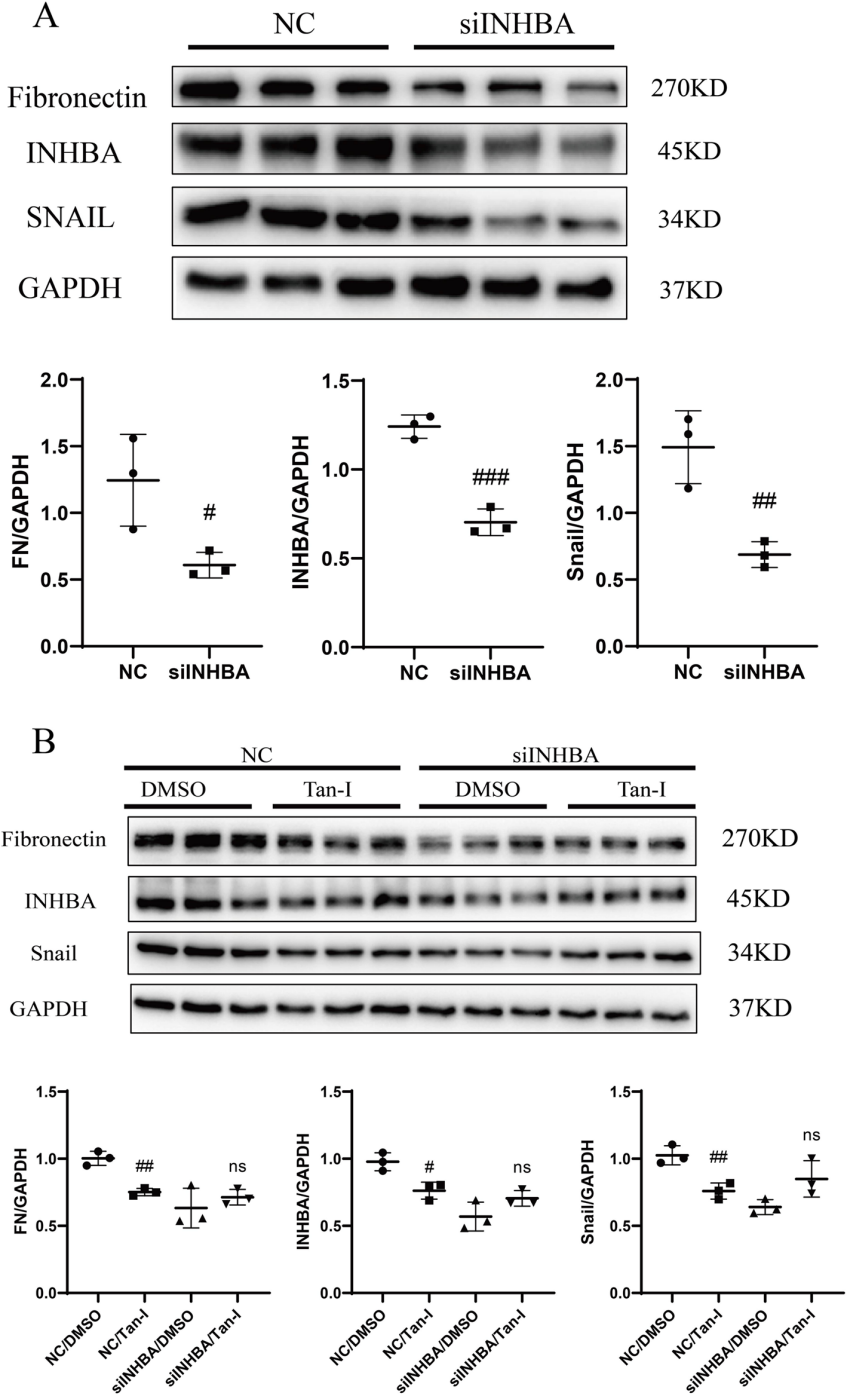
Tan-I inhibits fibrotic changes through INHBA. **A** NRK-49F cells were transfected with nonsense control (NC) or INHBA siRNA (siINHBA). After 24 h, cell lysates were collected and Western blot analysis was performed to measure the expression of INHBA, FN and Snail. Data represent mean ± SD. #p < 0.05 vs NC. ##*p* < 0.01 vs NC. ###*p* < 0.001 vs NC. *N* = 9 in each experimental group and one representative result of at least three independent experiments is shown. **B** NRF-49F cells were transfected with NC or siINHBA. On the second day, cells were treated 50 μM of Tan-I for another 24 h. Cell lysates were collected and Western blot analysis was performed to measure the expression of INHBA, FN and Snail. Data represents mean ± SD. ##*p* < 0.01 vs NC/DMSO. NS represents not significant. *N* = 9 in each experimental group and one representative result of at least three independent experiments is shown. Full-length blots are presented in Supplementary Fig. 5

INHBA was knocked down by siRNA in NRK-49F cells after 24 h transfection (Fig. 5A). As shown in Fig. 5A, the expression of FN and Snail were down-regulated in INHBA siRNA transfected NRK-49F cells.

Figure 5B shows that 50 μM Tan-I markedly reduced the expression of INHBA, FN, and Snail in non-sense control (NC)-siRNA transfected NRK-49F cells. The inhibitory effect of 50 μM Tan-I on the expression of FN and Snail was abolished in INHBA siRNA transfected NRK-49F cells (Fig. 5B).

## Discussion

In this study we determined the effect of Tan-I in renal fibrosis. We found that Tan-I dose-dependently inhibited the expression of EMT markers and phosphorylation of Smad3 in rat renal fibroblasts. In two different mouse models, we further showed that Tan-I attenuated renal fibrosis in UUO or AAN kidneys as shown by Masson staining and Western blotting analysis of fibrotic markers. Thus, we conclude that Tan-I inhibits renal fibrosis.

RNA-seq was performed to explore the underlining mechanism of anti-fibrotic effect of Tan-I. 88 differential expressed genes were identified between UUO/DMSO and UUO/Tan-I group, among which two genes (INHBA and BMP7) are involved in TGF-β signaling pathway. The predicted differential expression of BMP7 and INHBA were measured by qPCR, and only the expression of INHBA was significantly different between UUO/DMSO and UUO/Tan-I group. INHBA activates TGF-β signaling pathway and promotes EMT, however its role in renal fibrosis is current unknown [18, 19]. Since the importance and novelty of INHBA study in renal fibrosis, we further verified the expression of INHBA in protein levels. We found that INHBA is upregulated in two fibrotic mouse models, and Tan-I inhibited the expression of INHBA in UUO and AAN kidneys. Moreover, the direct inhibitory effect of Tan-I on INHBA expression was confirmed *in vitro*. We further showed that knockdown of INHBA reduced the expression of pro-fibrotic markers and abolished the inhibitory effect of Tan-I on the expression of pro-fibrotic markers in renal fibroblasts.

Multiple signaling pathways are regulated by Tan-I, among which nuclear factor erythroid 2 related factor 2 (Nrf2) signaling was identified as an important target of Tan-I [25–27]. Nrf2 inhibits inflammation, oxidative stress and protects mitochondria function in chronic kidney diseases [28]. Tan-I can directly activate Nrf2 as its potential agonist or indirectly activate Nrf2 through the Akt signaling pathway [25–27]. Thus, whether Tan-I inhibits renal interstitial fibrosis through Nrf2 warrants further study.

The methyltransferase Enhancer of zeste homolog 2 (EZH2) is an important epigenetic regulator and has been intensively studied in the field of renal diseases [29]. EZH2 and its downstream target histone H3 lysine 27 trimethylation (H3K27me3) are upregulated in mouse kidneys with renal interstitial fibrosis [30]. Blockage of EZH2 attenuates renal interstitial fibrosis through inhibition of epithelial-mesenchymal transition (EMT) transition and activation of anti-fibrotic signaling pathways such as PTEN and Smad7 [29–31]. A recent study showed that Tan-I is an inhibitor of EZH2, which directly binds to EZH2 and inhibits its enzymatic activity on histone methylation [32]. Thus, EZH2 mediated epigenetic gene regulation could be a mechanism underlying the role of Tan-I in renal fibrosis.

Inflammation is a major event underlying the progression of CKD [33]. The innate immunity pathways nuclear factor ĸB (NF-ĸB) is a pivotal driver of inflammation and activation of NF-ĸB triggers the section of many pro-inflammatory factors [34]. Inhibition of NF-ĸB attenuates renal fibrosis in animal models [35]. The anti-inflammatory effect of Tan-I has been reported recently [36]. Tan-I inhibits IL-1β-induced NF-ĸB activation in chondrocytes, a model for osteoarthritis [37]. Tan-I together with Tanshinone IIA/B attenuate LPS-induced inflammation in mastitis probably through the NF-κB signaling pathway [36]. Whether Tan-I inhibits renal fibrosis through the NF-κB signaling pathway should be investigated in the future.

## Conclusion

We conclude that Tan-I attenuates fibrosis in fibrotic kidneys through down-regulation of INHBA. Our study suggests that Tan-I could be a novel and effective approach to treat CKD patients with renal interstitial fibrosis.

## Supplementary Information

**Additional file 1.** The original images of Western blot assay in figure 1

**Additional file 2.** The original images of Western blot assay in figure 2

**Additional file 3.** The original images of Western blot assay in figure 3

**Additional file 4.** The original images of Western blot assay in figure 4

**Additional file 5.** The original images of Western blot assay in figure 5

## Data Availability

The datasets used and/or analyzed during the current study are available from the corresponding author on reasonable request.

## Abbreviations

Tan-I: Tanshinone I
NRK-49F: Rat renal fibroblasts
UUO: Unilateral ureteral obstructionn
AAI: Aristolochic acid I
INHBA: Inhibin beta-A
CKD: Chronic kidney disease
ECM: Ext-racellular matrix
TGF-β: Transforming growth factor beta
EMT: Epithelial mesenchy-mal transition
FN: Fibronectin
α-SM-A: α Smooth muscle actin
AAN: Aristolochic acid nephropathy
Col-I: Collagen-I
pSmad3: Phosphorylated Smad-3
SD: Standard deviati-on
KEGG: Kyoto Encyclopedia of Genes and Genome-s
a-ctivin A: A subunit of inhibin A
GAPDH: Glyceraldehyde-3-phosphate dehydrogenase
