# Supplementary material for: Tanshinone I attenuates fibrosis in fibrotic kidneys through down-regulation of inhibin beta-A
Source: BMC Complement Med Ther. 2022 Apr 19;22:110. doi: 10.1186/s12906-022-03592-3 (PMC9020026; doi:10.1186/s12906-022-03592-3)

repeat1

cropped

mk	Tan-I		Tan-I		Tan-I		Tan-I		mk
μM	0	0	0.5	0.5	5	5	50	50	

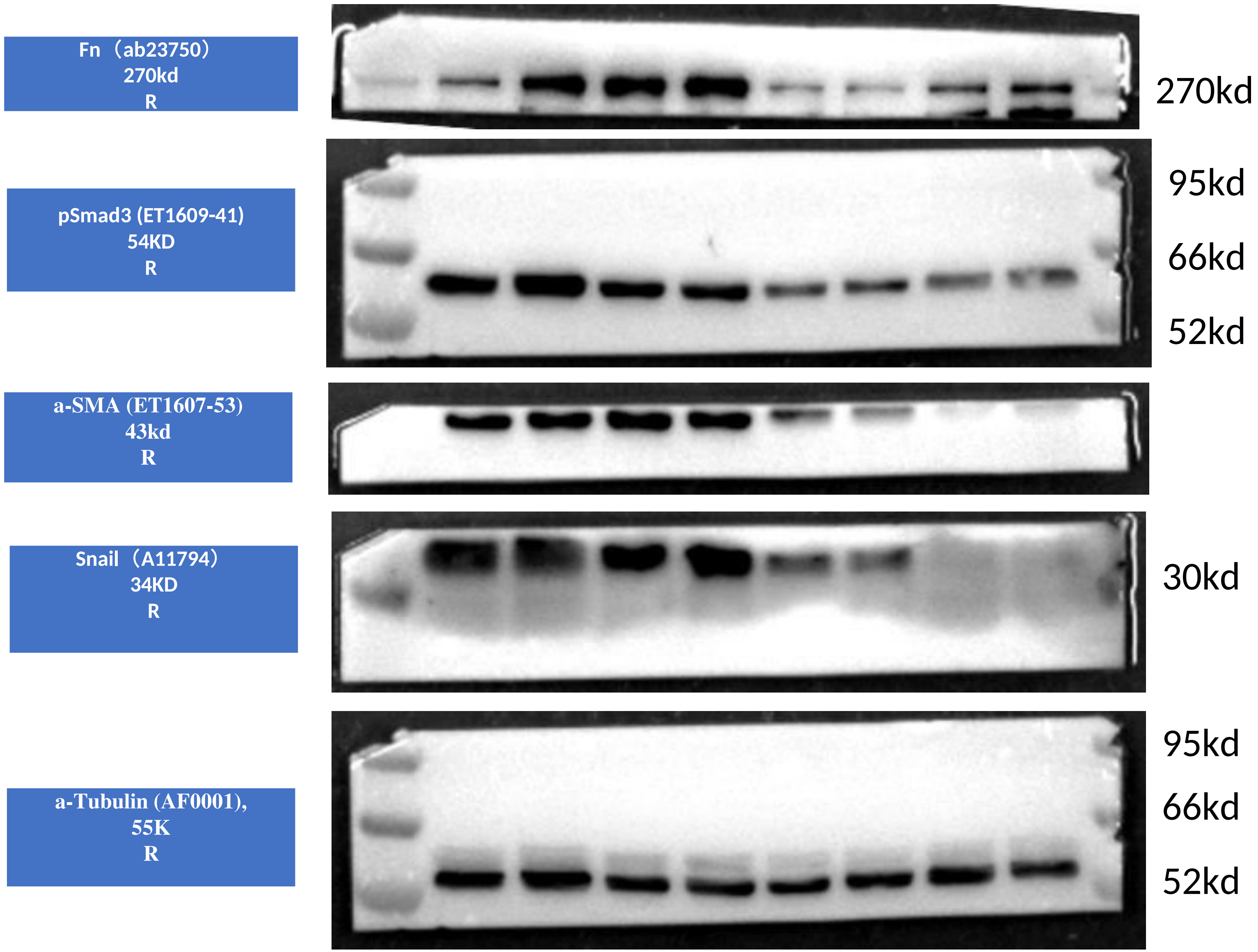

Figure 1. Tanshinone I (Tan-I) inhibits fibrotic changes of rat renal fibroblasts

Rat renal fibroblasts (NRK-49F) were starved for 24h and followed by 24h treatment with different concentration (0.5, 5, 50 μM) of Tan-I. The expression of fibronectin (FN), α-smooth muscle actin (α-SMA), Snail and phosphorylation of Smad3 (pSmad3) were analyzed by Western blotting and then quantified.

repeat1

original

merged

Fn（ab23750）
270kd
R

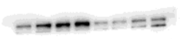

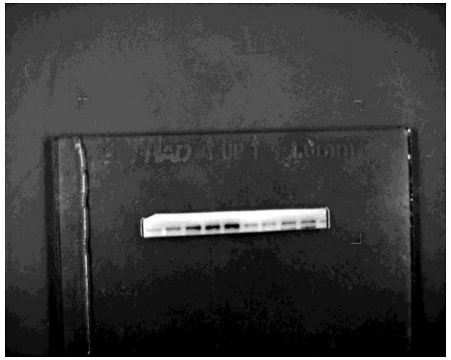

pSmad3（ET1609-41)，54KD，R

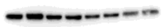

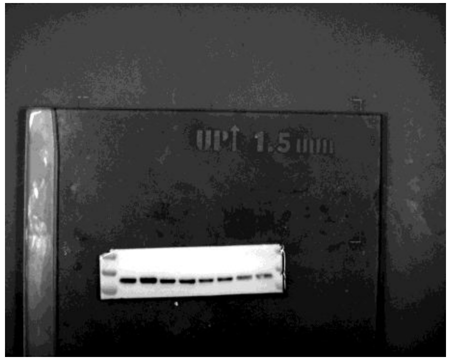

a-SMA (ET1607-53),R,43kd

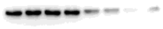

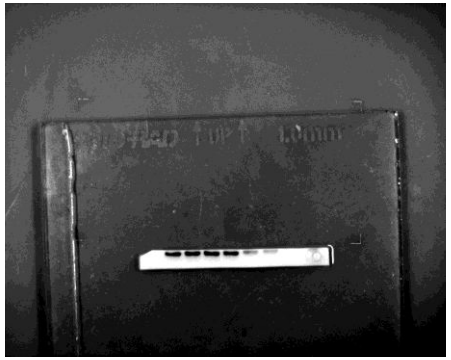

Snail（A11794）
34KD
R

original

merged

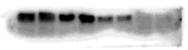

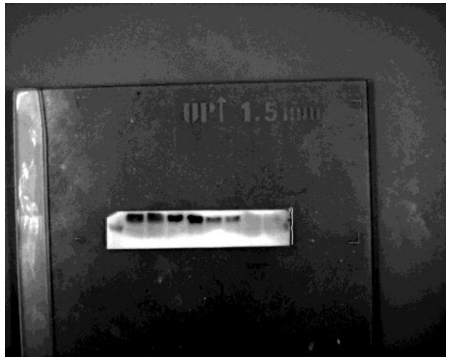

a-Tubulin (AF0001), R, 55KD

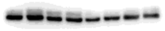

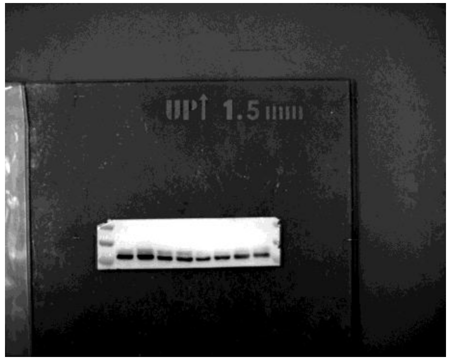

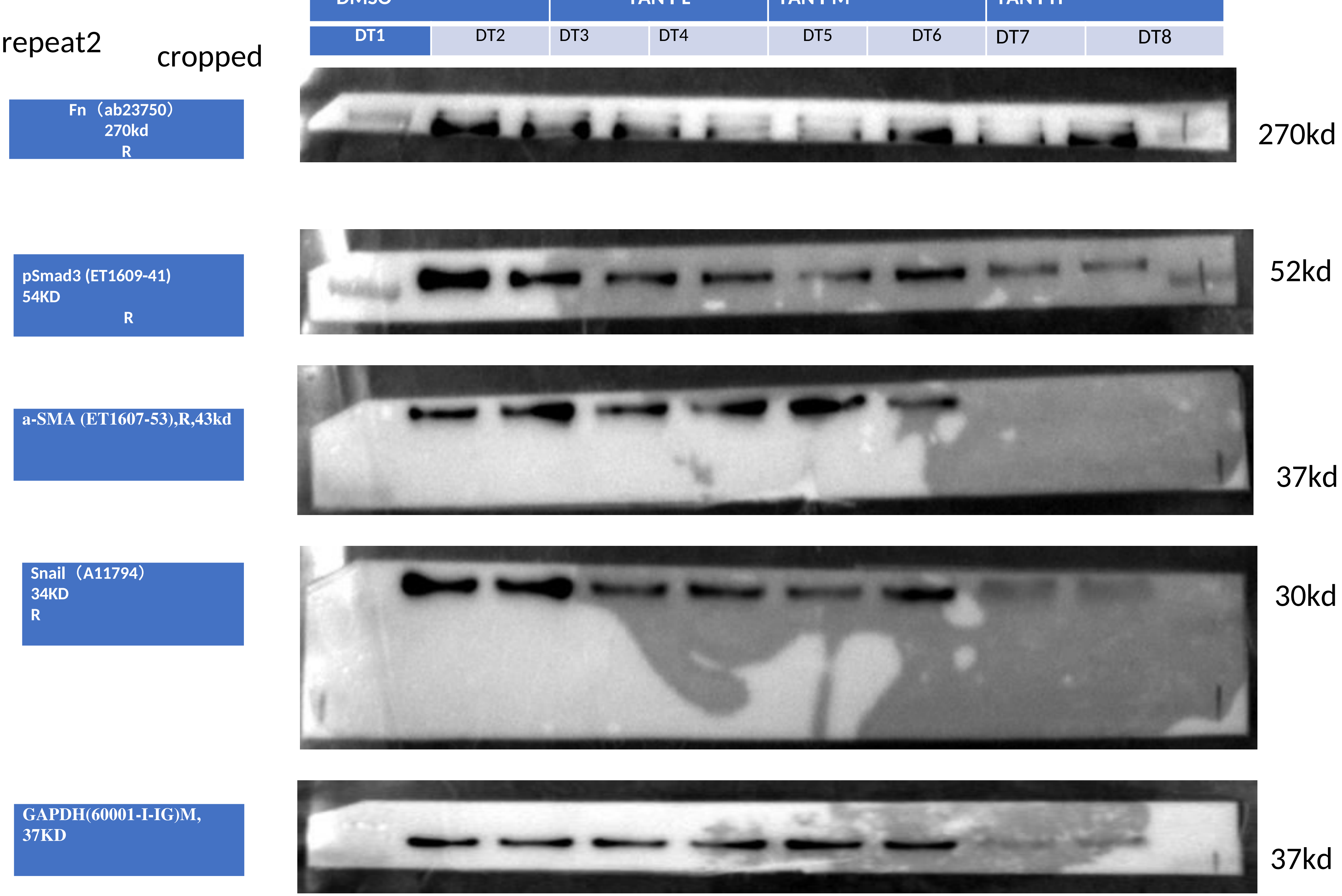
repeat2
cropped
DT1
DT2
DT3
DT4
DT5
DT6
DT7
DT8
Fn（ab23750）
270kd
R
270kd
pSmad3 (ET1609-41)
54KD
R
52kd
a-SMA (ET1607-53),R,43kd
37kd
Snail（A11794）
34KD
R
30kd
GAPDH(60001-I-IG)M,
37KD
37kd

repeat2

Fn

original

merged

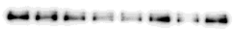

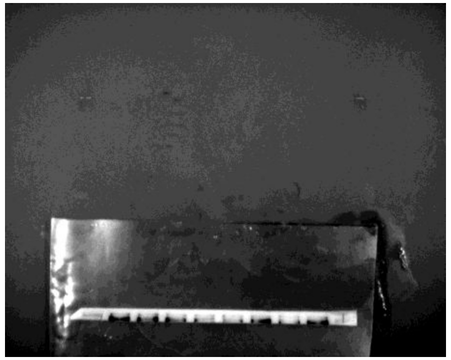

psmad3

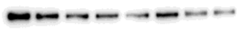

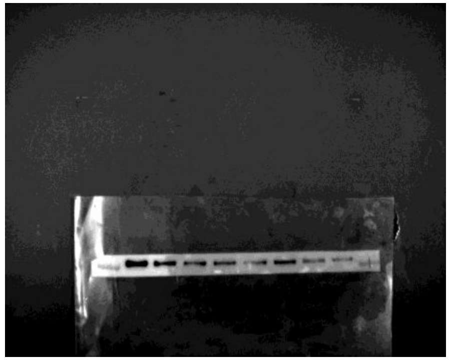

α-sm

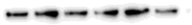

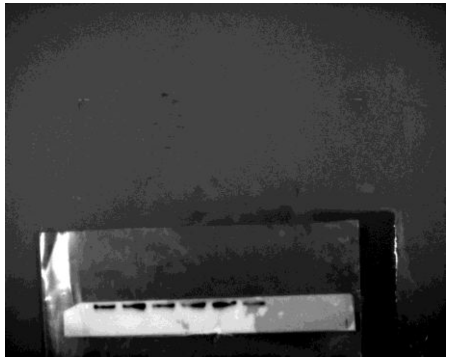

original

merged

snail

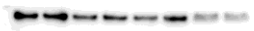

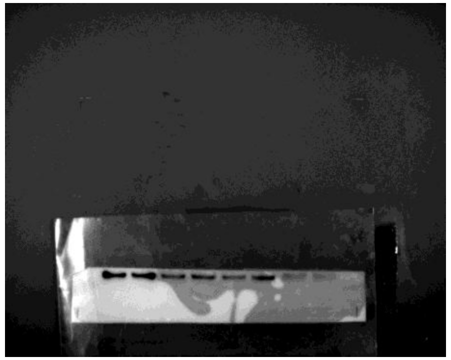

Gapdh

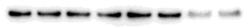

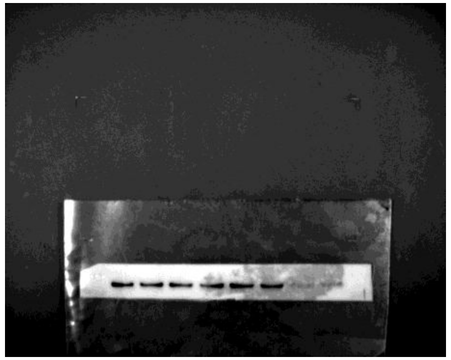

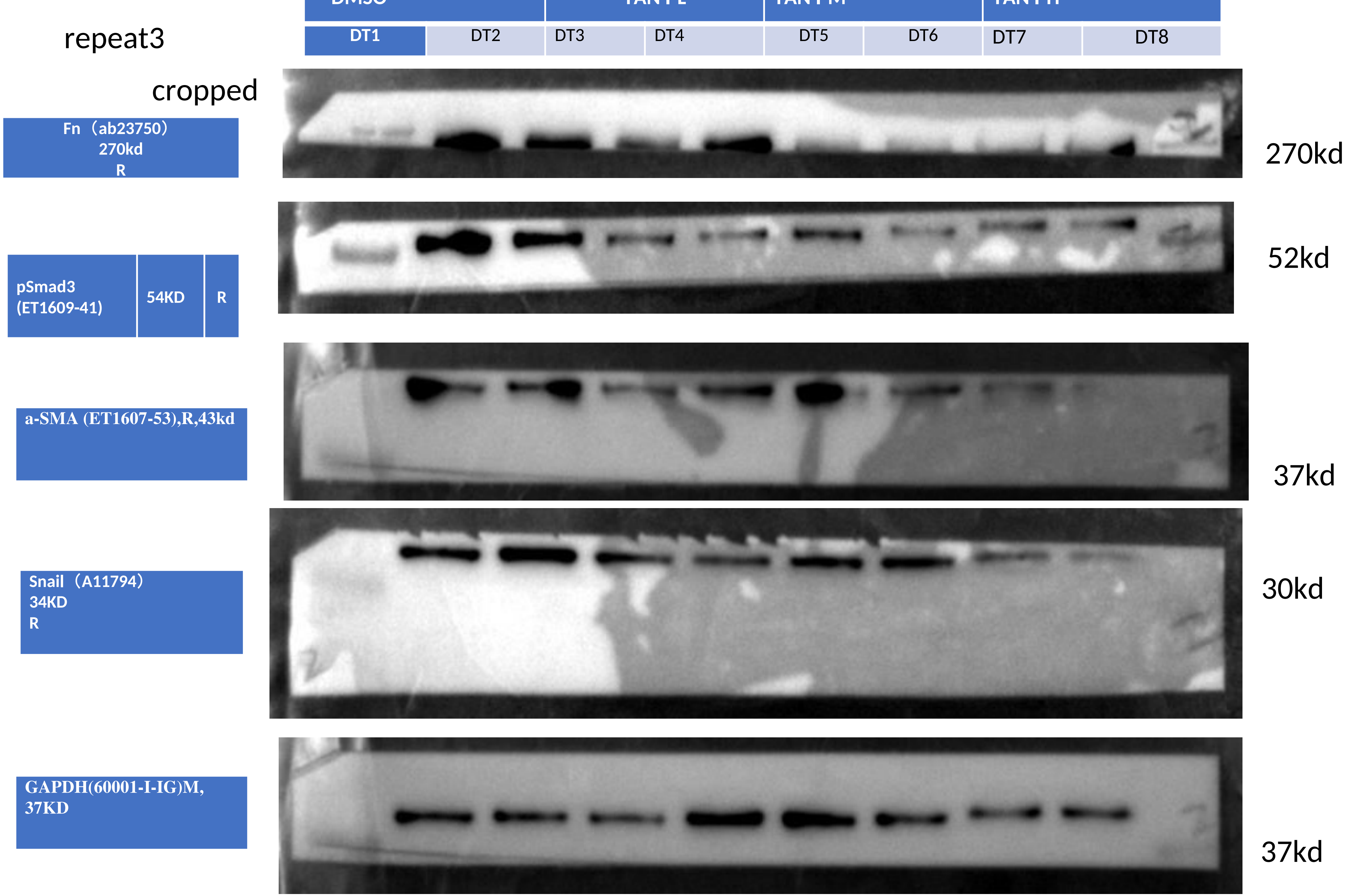

DT1
DT2
DT3
DT4
DT5
DT6
DT7
DT8
repeat3
cropped
Fn（ab23750）
270kd
R
270kd
pSmad3
(ET1609-41)
54KD
R
52kd
a-SMA (ET1607-53),R,43kd
37kd
Snail（A11794）
34KD
R
30kd
GAPDH(60001-I-IG)M,
37KD
37kd

Repeat3

original

merged

fn

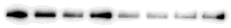

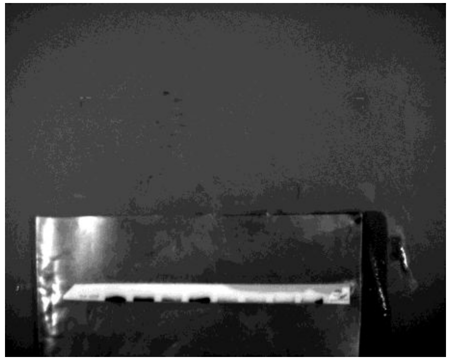

psmad3

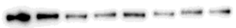

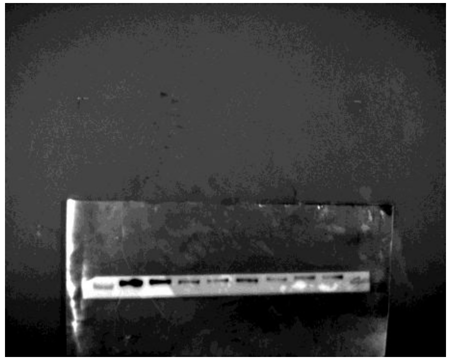

α-sma

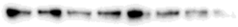

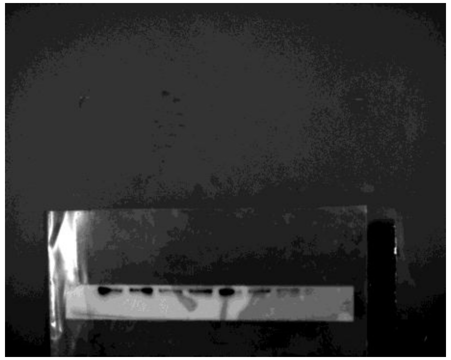

original

merged

Snail

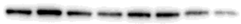

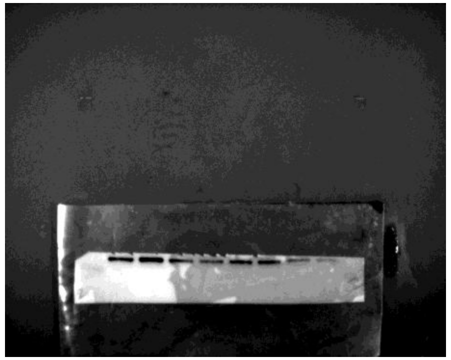

GAPDH

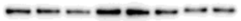

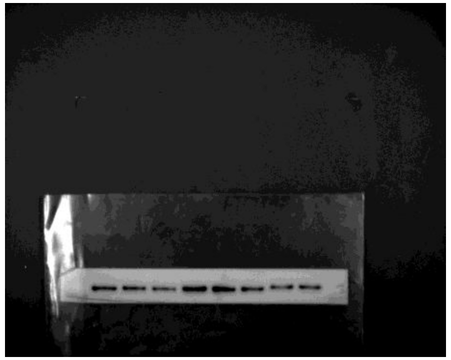

Supplement: Supplementary file 1 — Additional file 1. The original images of Western blot assay in figure 1 [file 12906_2022_3592_MOESM1_ESM.pdf]
