# Supplementary material for: Tanshinone I attenuates fibrosis in fibrotic kidneys through down-regulation of inhibin beta-A
Source: BMC Complement Med Ther. 2022 Apr 19;22:110. doi: 10.1186/s12906-022-03592-3 (PMC9020026; doi:10.1186/s12906-022-03592-3)

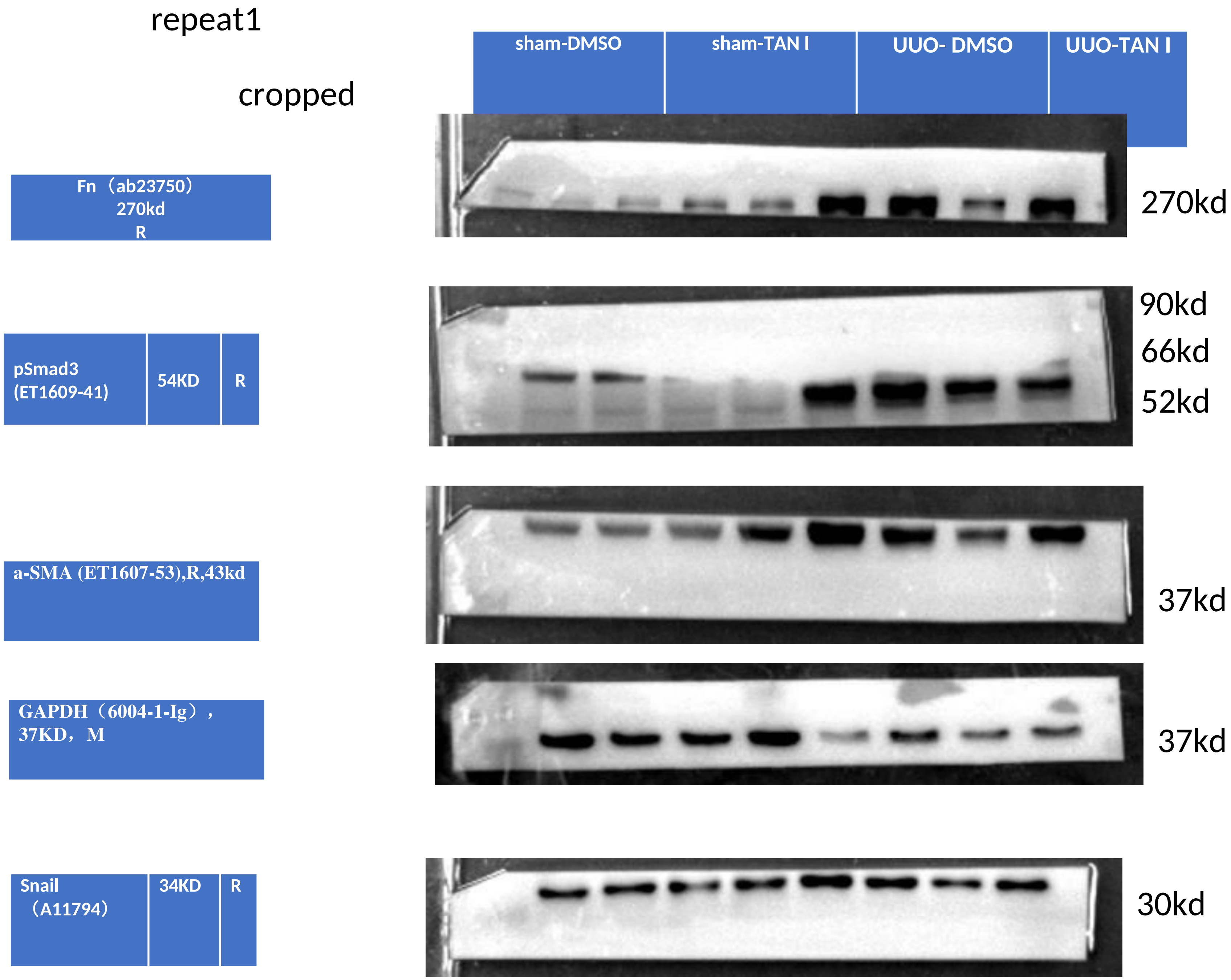

Figure 2. Tan-I mitigates renal fibrosis in UUO mice

The expression of FN, α-SMA, Snail and pSmad3 were analyzed by Western blotting and then quantified.

Fn（ab23750）
270kd
R

original

merged

repeat1

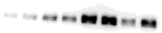

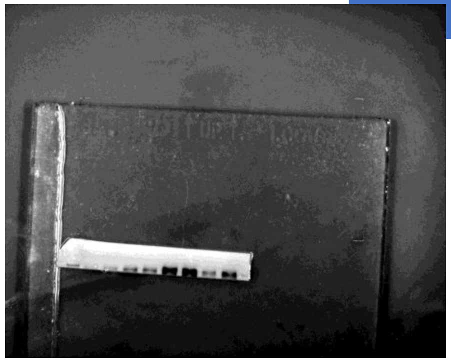

pSmad3
(ET1609-41)

54KD

R

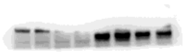

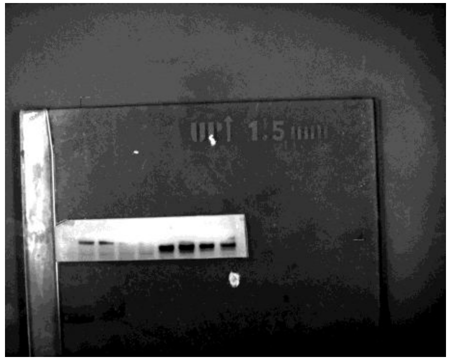

a-SMA (ET1607-53),R,43kd

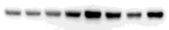

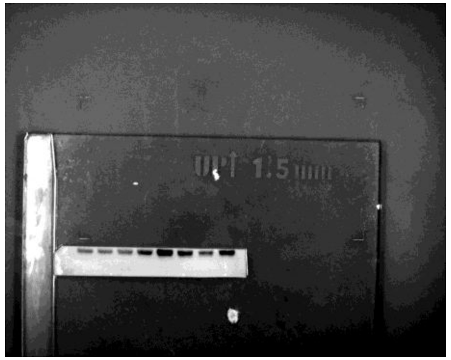

Snail
（A11794）

34KD

R

original

merged

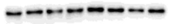

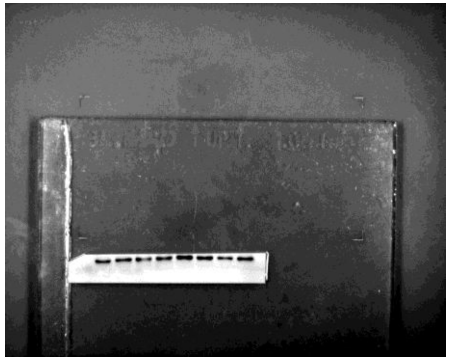

GAPDH（6004-1-Ig），
37KD，M

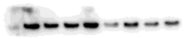

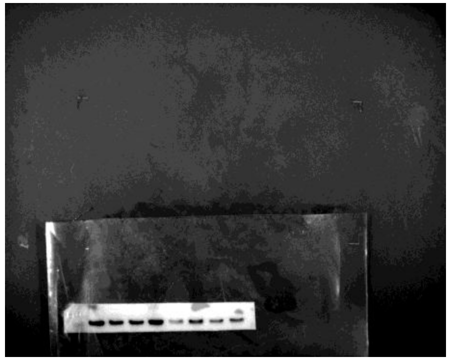

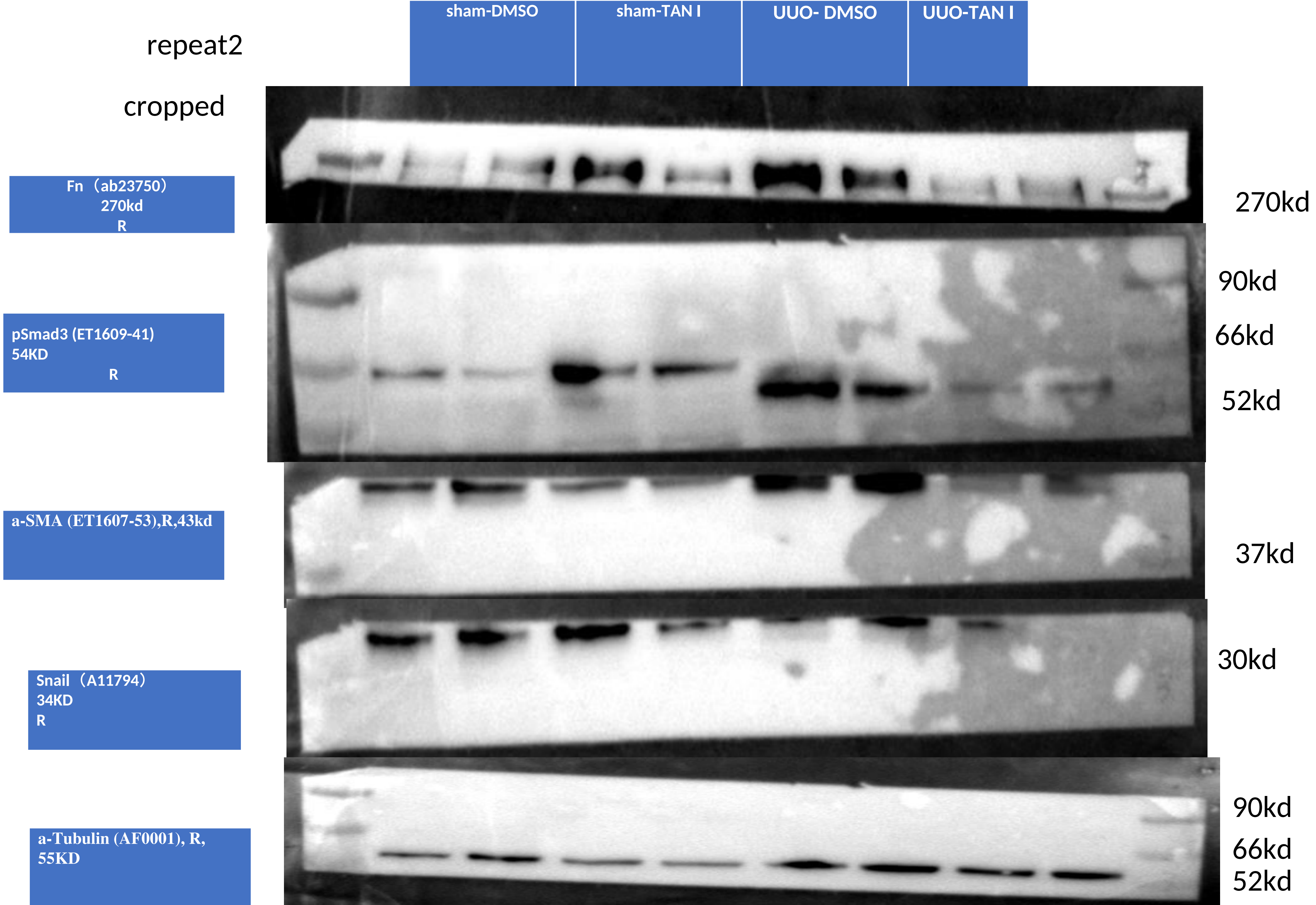
repeat2
cropped
sham-DMSO
sham-TAN I
UUO- DMSO
UUO-TAN I
Fn（ab23750）
270kd
R
270kd
pSmad3 (ET1609-41)
54KD
R
90kd
66kd
52kd
a-SMA (ET1607-53),R,43kd
37kd
Snail（A11794）
34KD
R
30kd
a-Tubulin (AF0001), R,
55KD
90kd
66kd
52kd

Fn（ab23750）
270kd
R

original

merged

repeat2

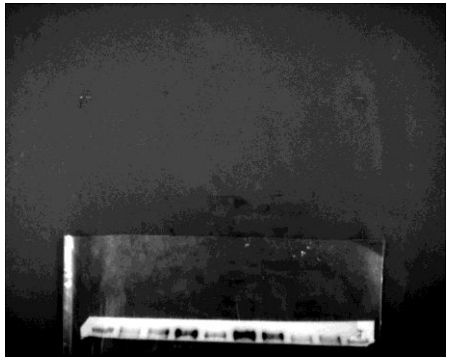

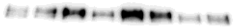

pSmad3 (ET1609-41)
54KD
R

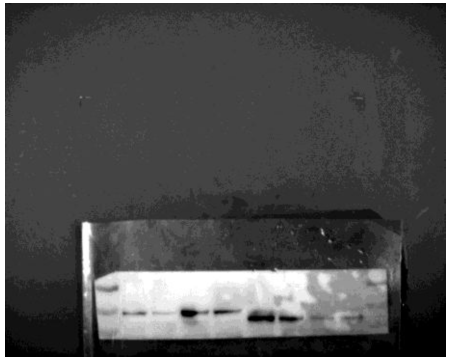

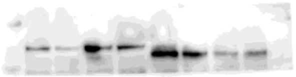

a-SMA (ET1607-53),R,43kd

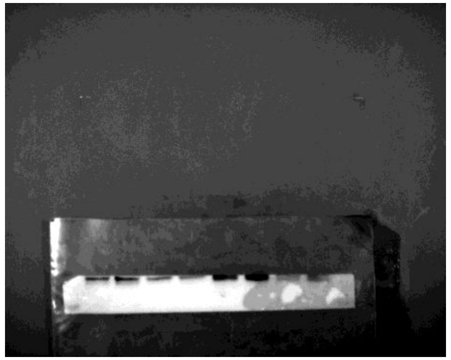

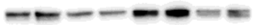

Snail（A11794）
34KD
R

original

merged

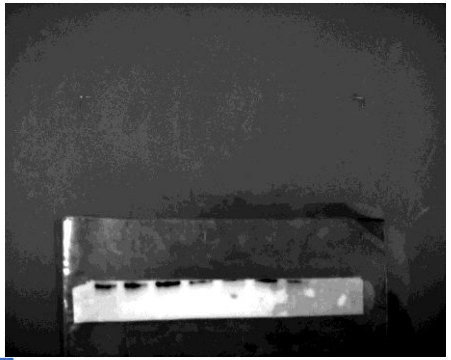

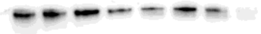

a-Tubulin (AF0001), R,
55KD

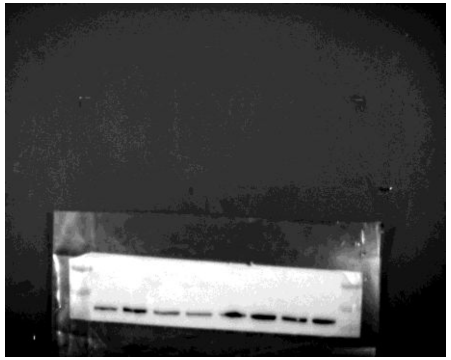

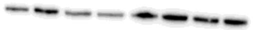

repeat3

cropped

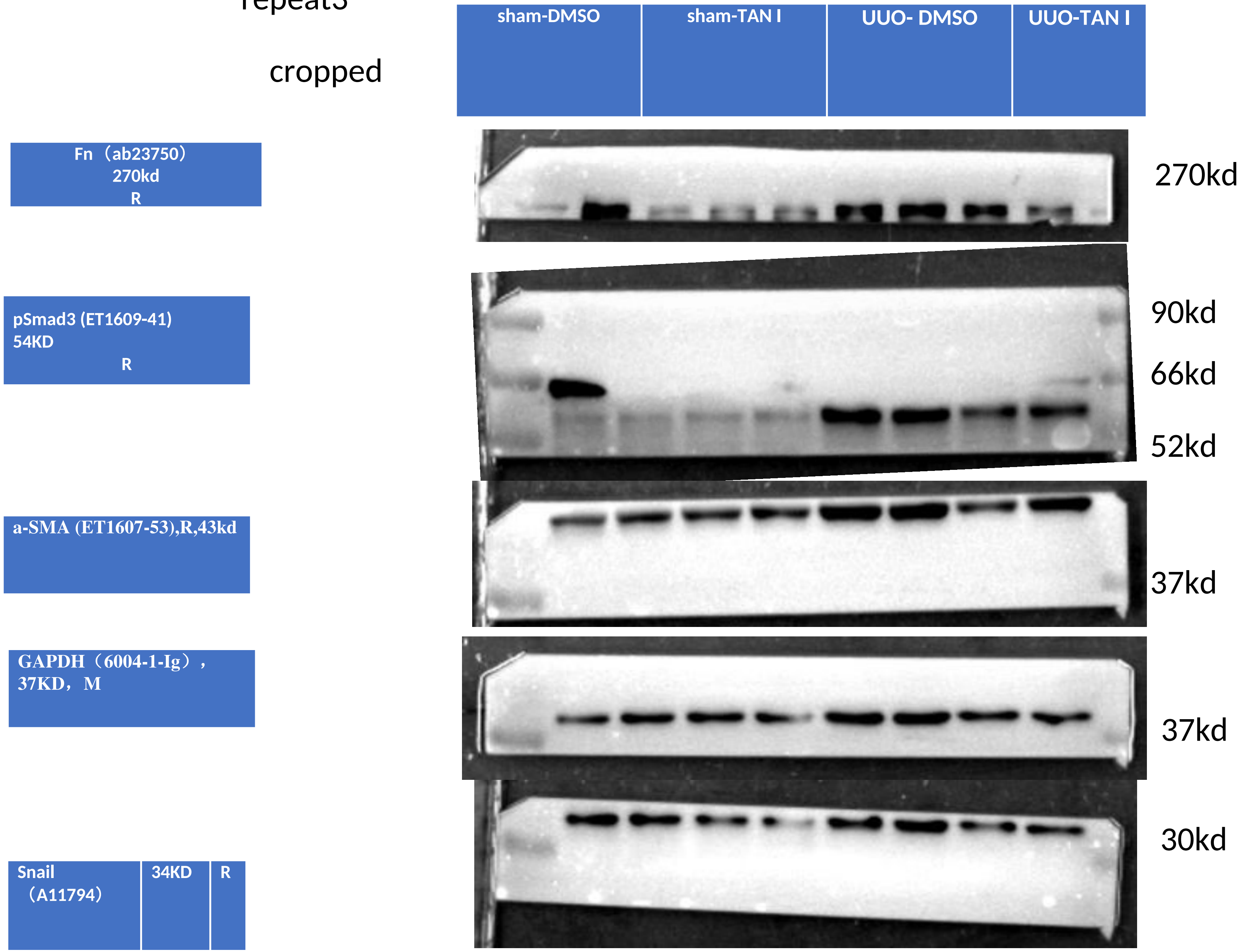

Fn（ab23750）
270kd
R

original

merged

repeat3

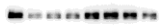

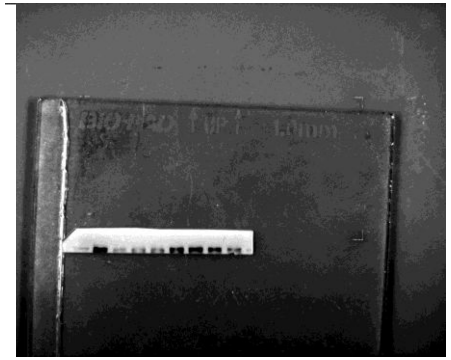

pSmad3 (ET1609-41)
54KD
R

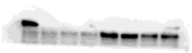

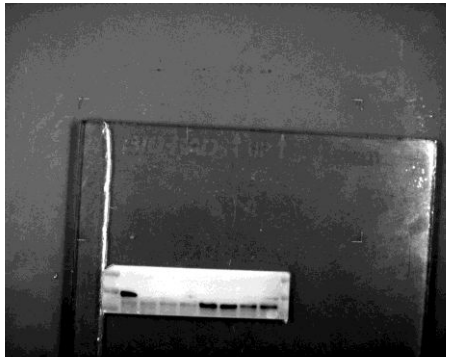

a-SMA (ET1607-53),R,43kd

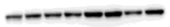

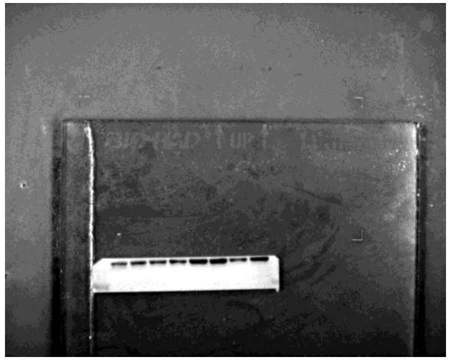

Snail（A11794）	34KD	R

original

merged

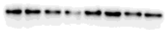

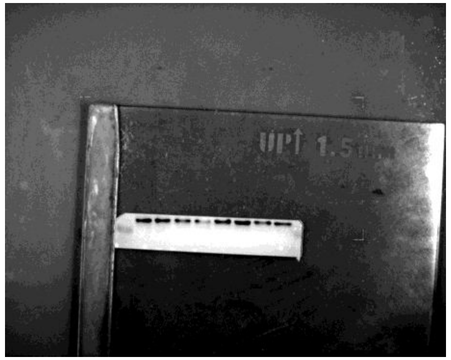

GAPDH（6004-1-Ig），
37KD，M

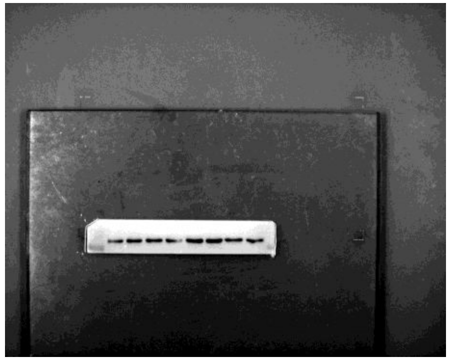

Supplement: Supplementary file 2 — Additional file 2. The original images of Western blot assay in figure 2 [file 12906_2022_3592_MOESM2_ESM.pdf]
