# Supplementary material for: Tanshinone I attenuates fibrosis in fibrotic kidneys through down-regulation of inhibin beta-A
Source: BMC Complement Med Ther. 2022 Apr 19;22:110. doi: 10.1186/s12906-022-03592-3 (PMC9020026; doi:10.1186/s12906-022-03592-3)

Figure 3. Tan-I ameliorates renal fibrosis in AAN mice

The expression of FN, α-SMA, Snail1 and pSmad3 were analyzed by Western blotting and then quantified.

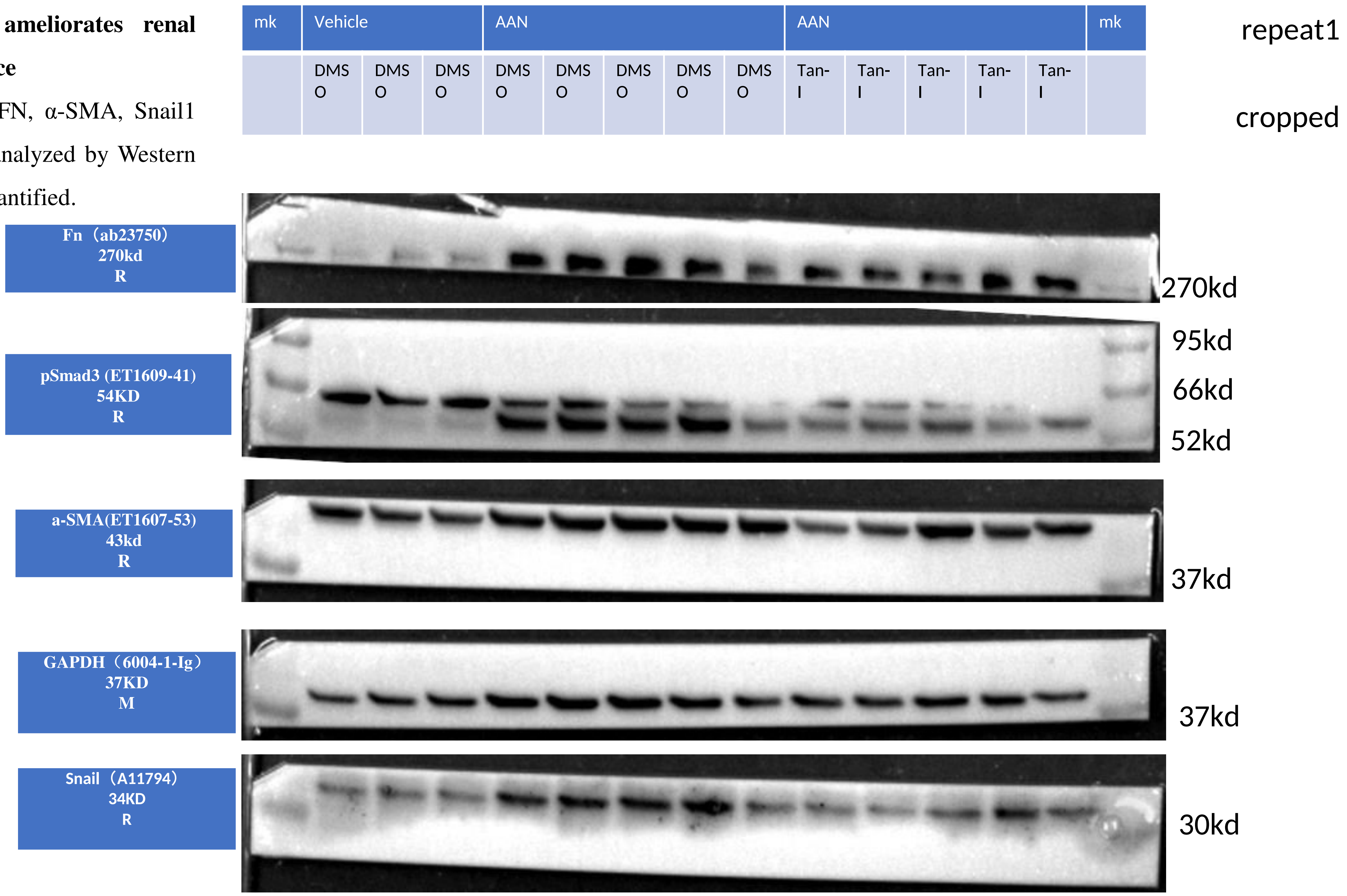

Fn（ab23750）
270kd
R

original

merged

repeat1

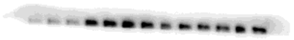

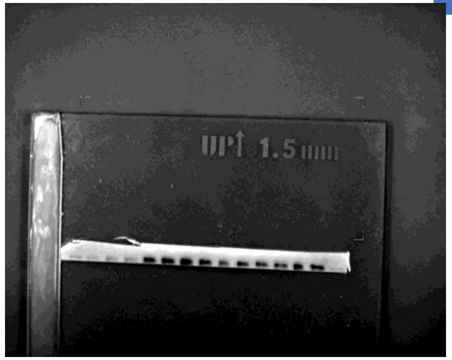

pSmad3 (ET1609-41) | 54KD | R

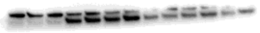

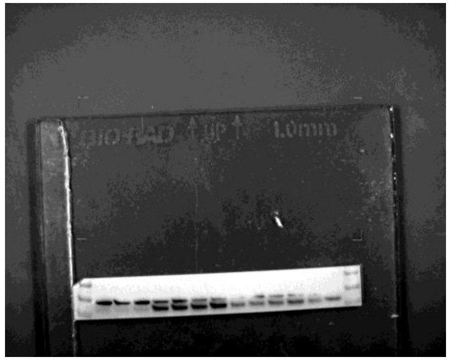

a-SMA (ET1607-53),R,43kd

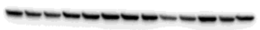

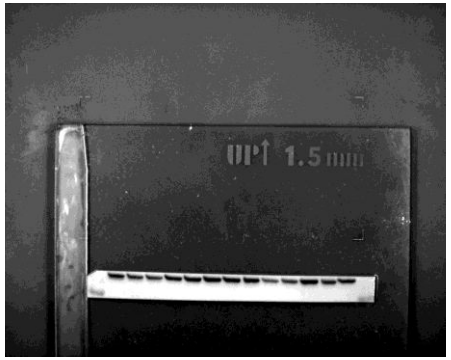

Snail（A11794） | 34KD | R

original

merged

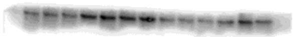

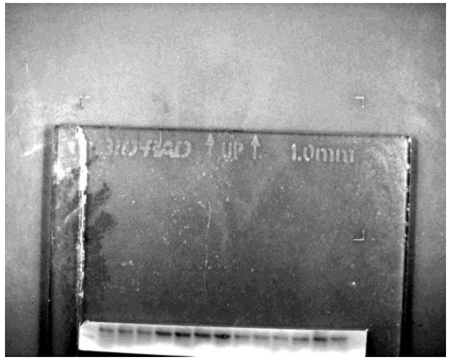

GAPDH（6004-1-Ig）
37KD
M

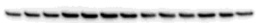

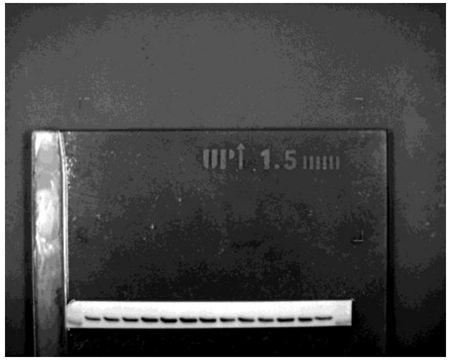

repeat2

cropped

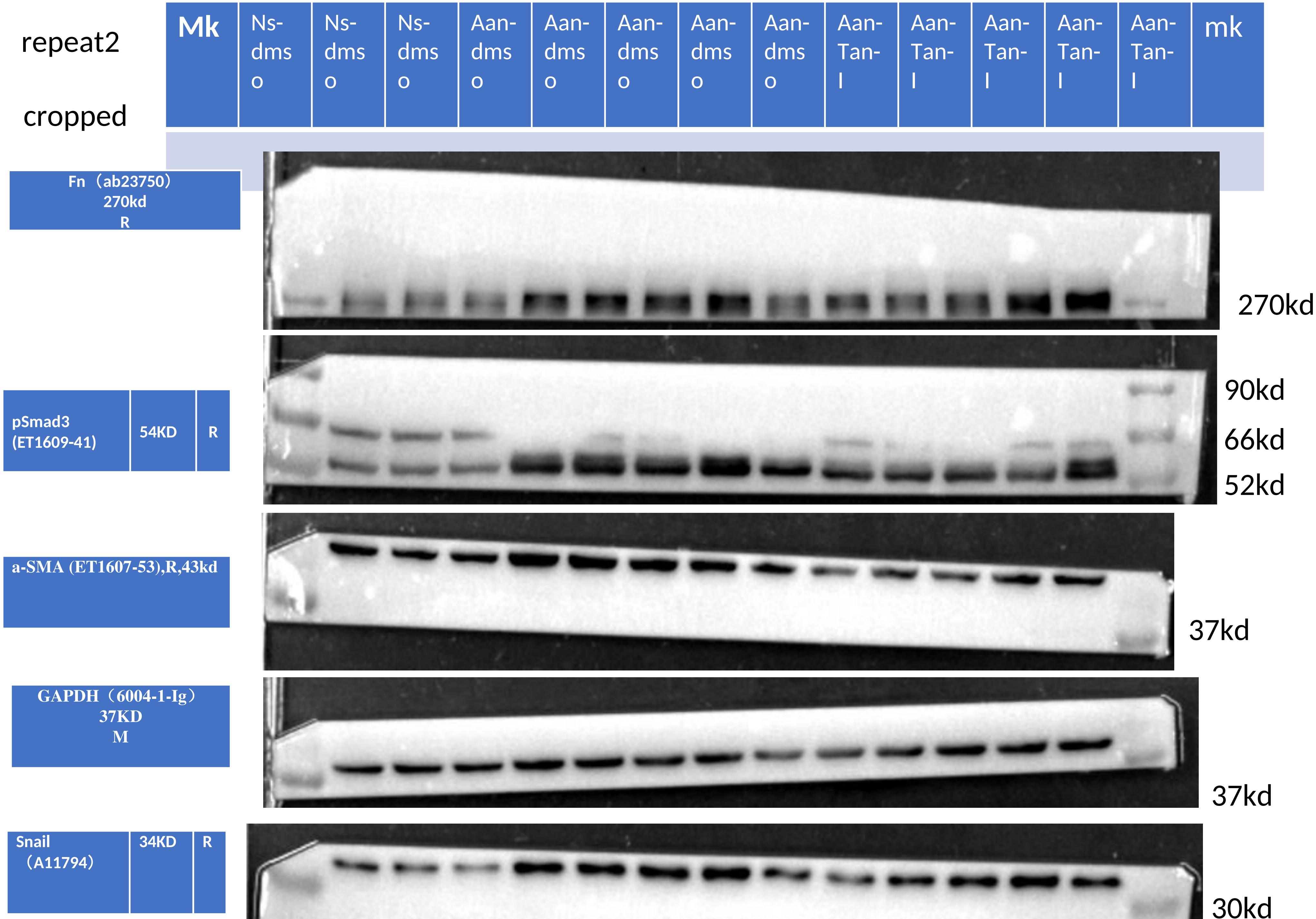

Fn（ab23750）
270kd
R

original

merged

repeat2

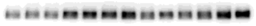

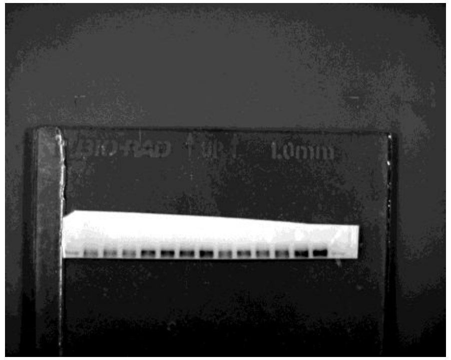

pSmad3 (ET1609-41)	54KD	R

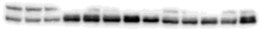

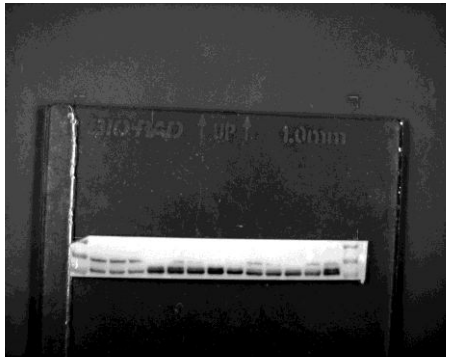

a-SMA (ET1607-53),R,43kd

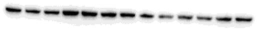

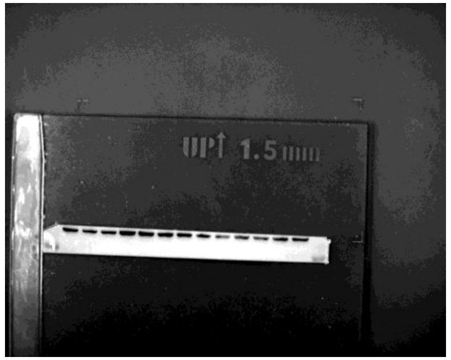

Snail （A11794）	34KD	R

original

merged

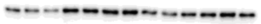

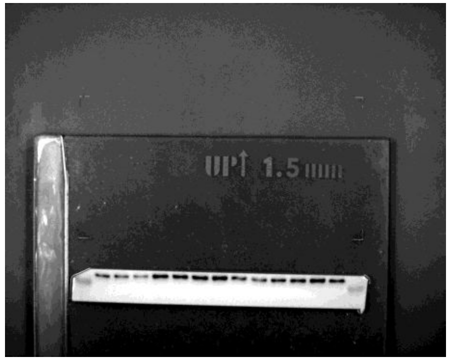

GAPDH（6004-1-Ig）
37KD
M

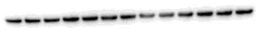

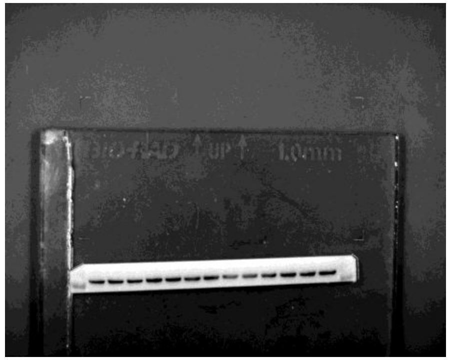

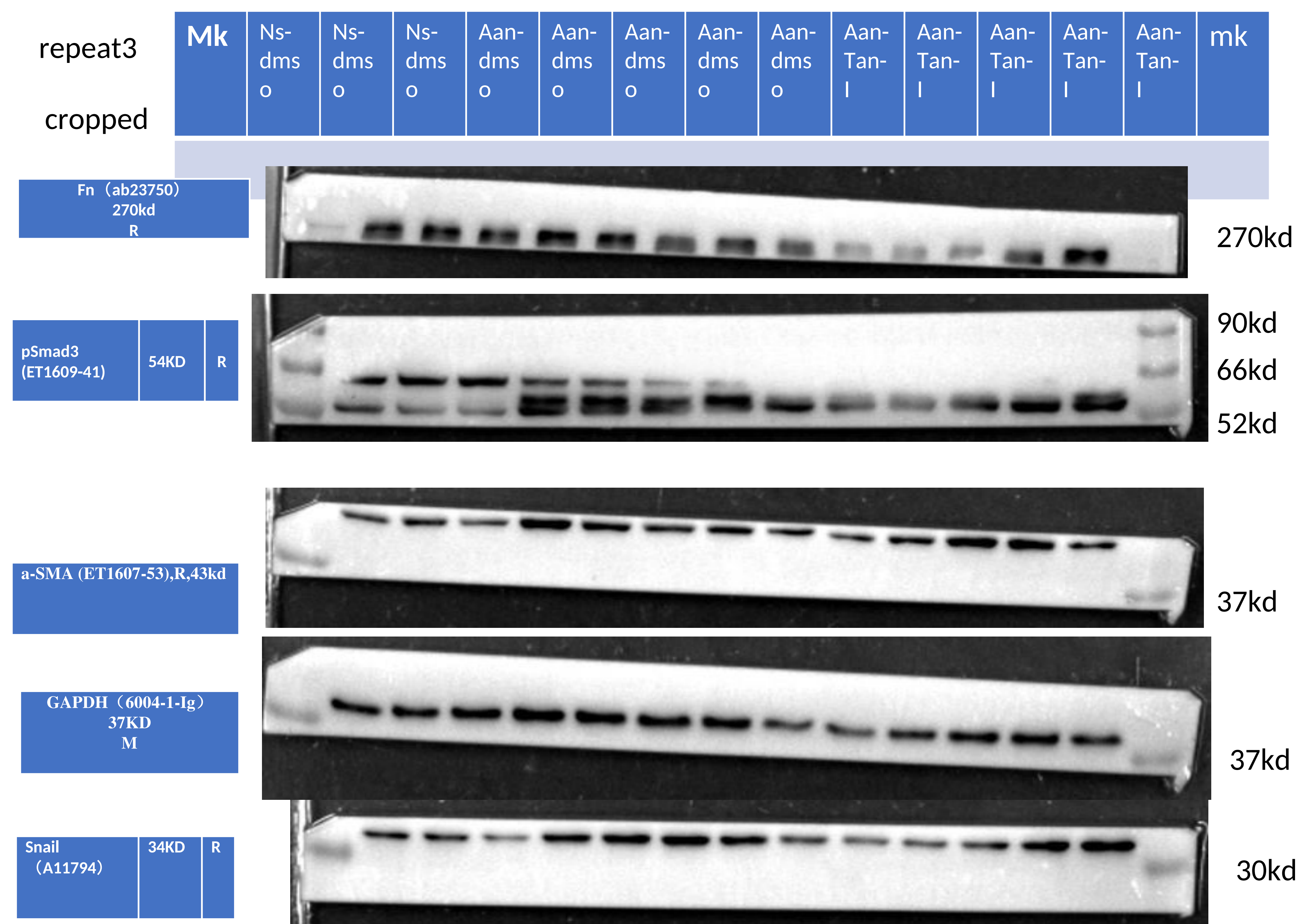
repeat3
cropped
Mk
Ns-dmso
Ns-dmso
Ns-dmso
Aan-dmso
Aan-dmso
Aan-dmso
Aan-dmso
Aan-dmso
Aan-Tan-I
Aan-Tan-I
Aan-Tan-I
Aan-Tan-I
Aan-Tan-I
mk
Fn（ab23750）270kd R
270kd
pSmad3 (ET1609-41)
54KD
R
90kd
66kd
52kd
a-SMA (ET1607-53),R,43kd
37kd
GAPDH（6004-1-Ig）37KD M
37kd
Snail（A11794）
34KD
R
30kd

Fn（ab23750）
270kd
R

original

merged

repeat3

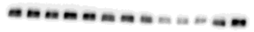

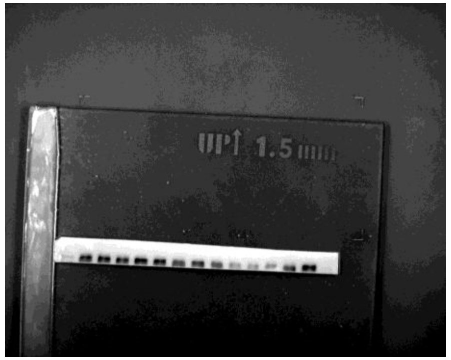

pSmad3
(ET1609-41)

54KD

R

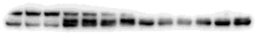

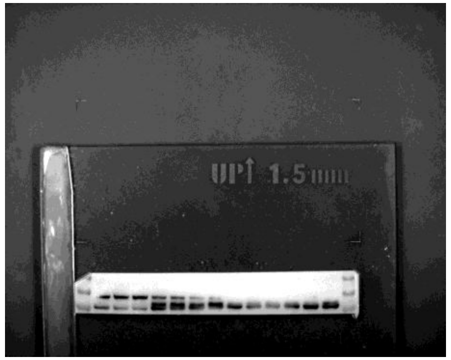

a-SMA (ET1607-53),R,43kd

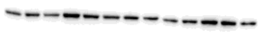

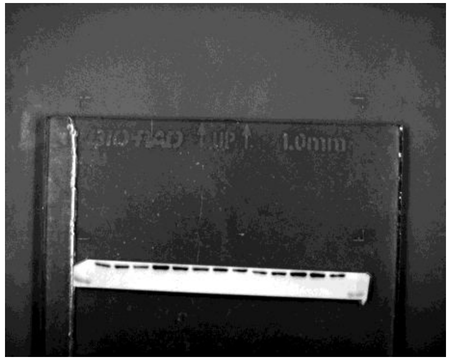

original

merged

Snail
（A11794）

34KD

R

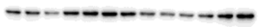

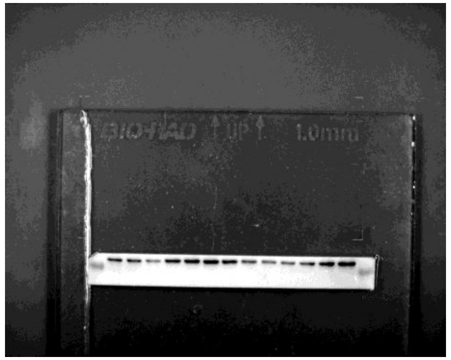

GAPDH（6004-1-Ig）
37KD
M

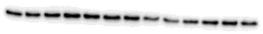

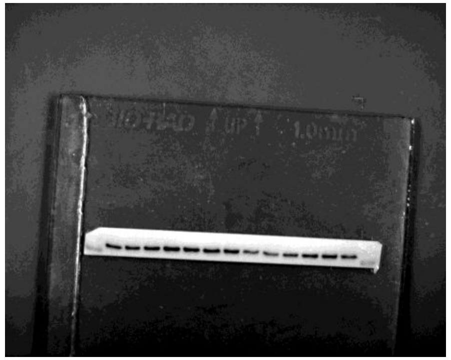

Supplement: Supplementary file 3 — Additional file 3. The original images of Western blot assay in figure 3 [file 12906_2022_3592_MOESM3_ESM.pdf]
