# Supplementary material for: Tanshinone I attenuates fibrosis in fibrotic kidneys through down-regulation of inhibin beta-A
Source: BMC Complement Med Ther. 2022 Apr 19;22:110. doi: 10.1186/s12906-022-03592-3 (PMC9020026; doi:10.1186/s12906-022-03592-3)

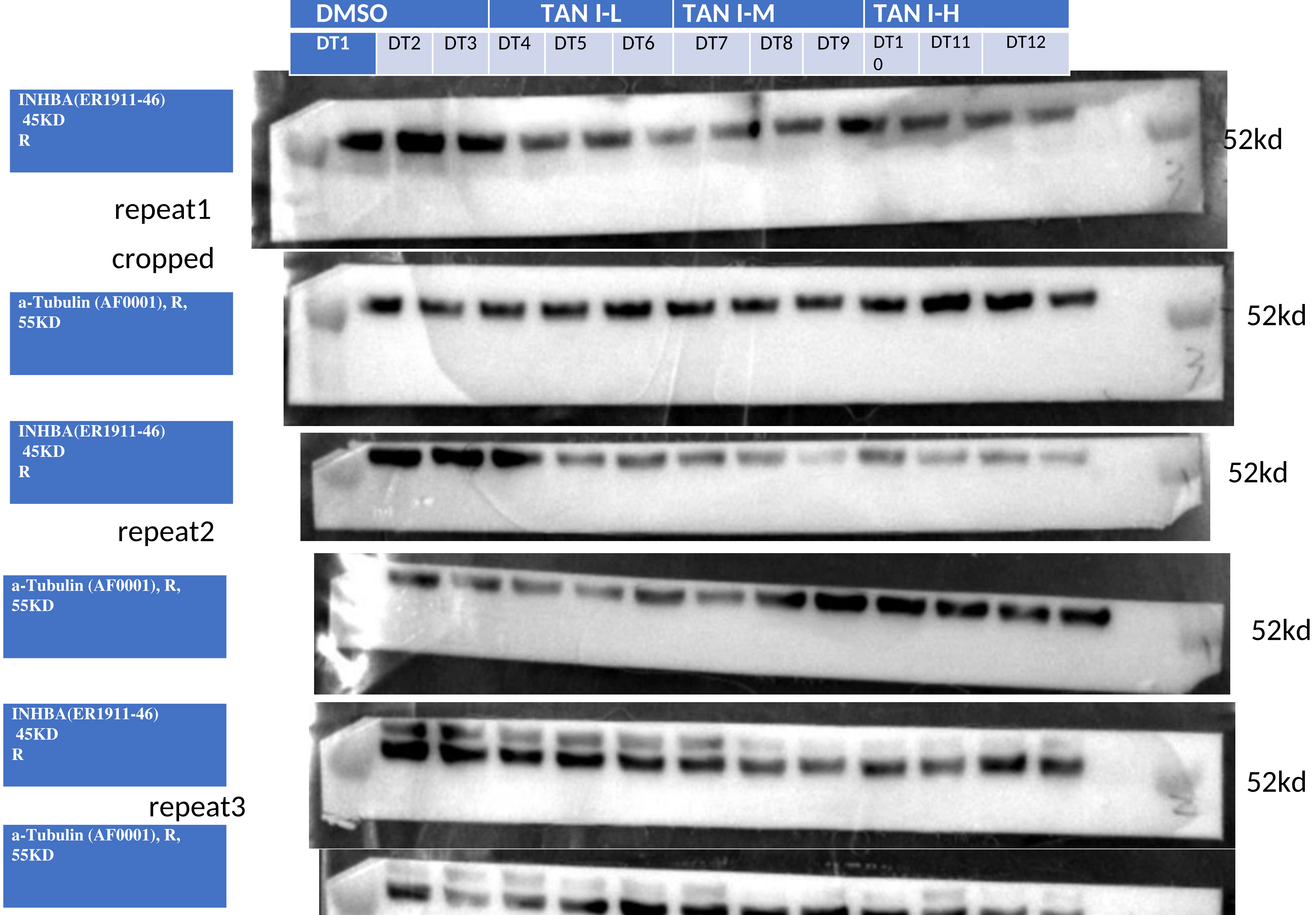

NRK-49F cells were starved for 24h and followed by 24h treatment with different concentration (0.5, 5, 50 μM) of Tan-I. The expression of inhibin beta-A (INHBA) was analyzed by Western blotting and then quantified.

original

merged

INHBA(ER1911-46)
45KD
R

repeat1

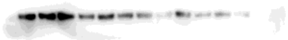

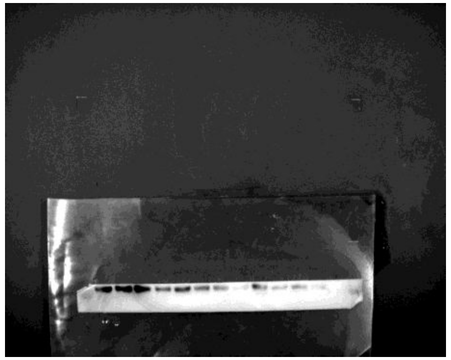

INHBA(ER1911-46)
45KD
R

repeat2

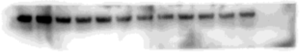

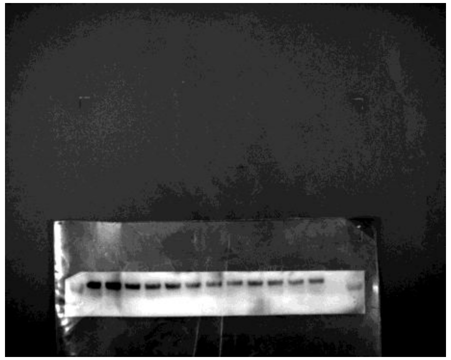

INHBA(ER1911-46)
45KD
R

repeat3

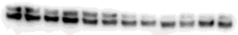

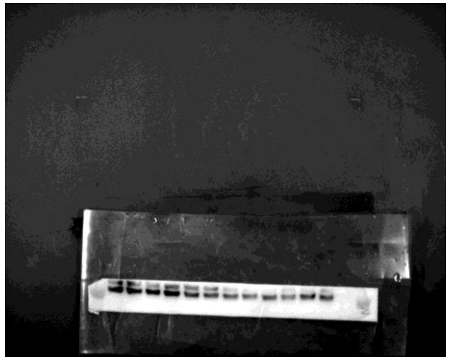

a-Tubulin (AF0001),
R, 55KD

original

merged

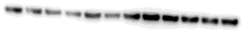

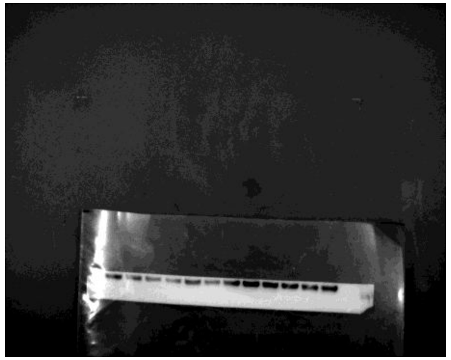

a-Tubulin (AF0001),
R, 55KD

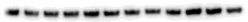

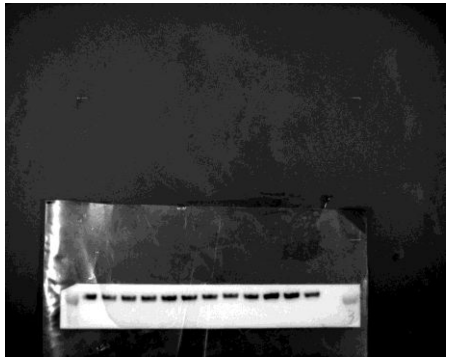

a-Tubulin (AF0001),
R, 55KD

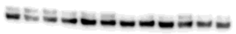

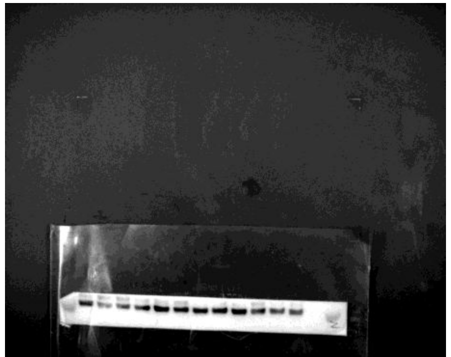

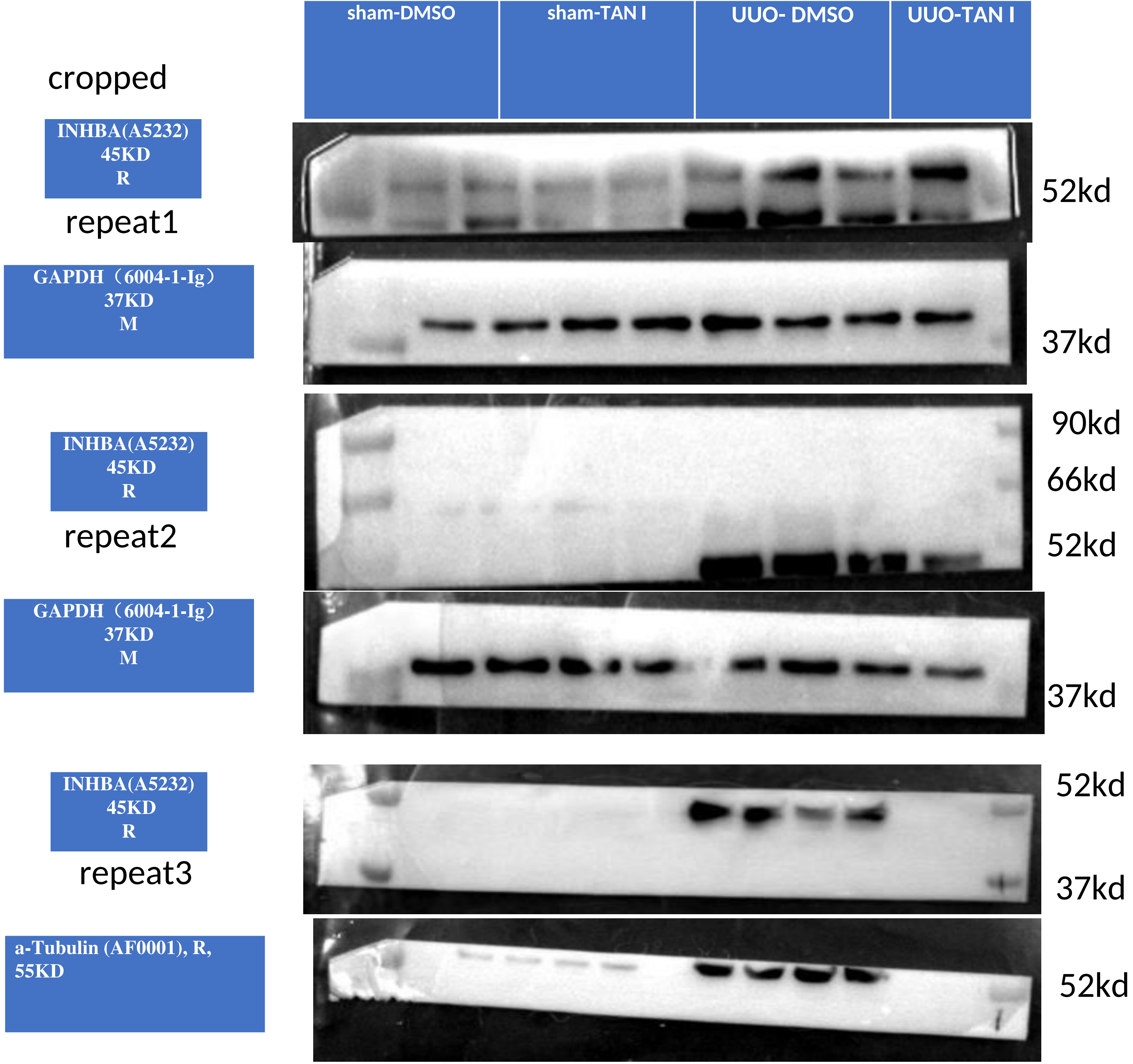

The expression of INHBA in sham or UUO kidneys was analyzed by Western blotting and then quantified.

original

merged

INHBA(A5232)
45KD
R

repeat1

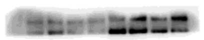

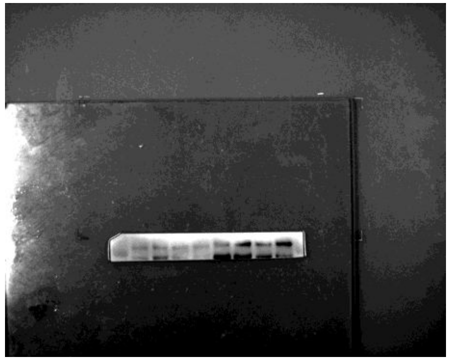

INHBA(A5232)
45KD
R

repeat2

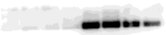

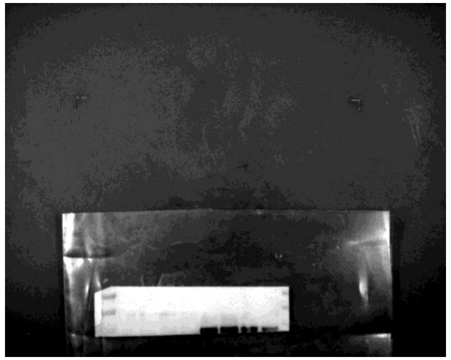

INHBA(A5232)
45KD
R

repeat3

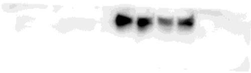

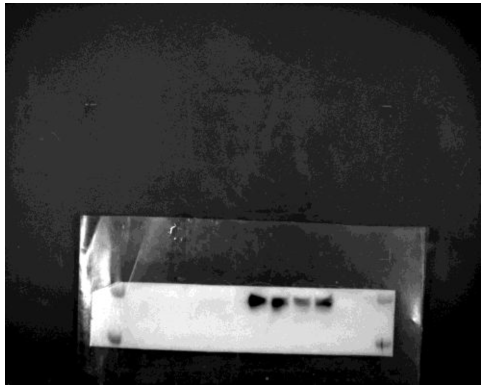

GAPDH（6004-1-Ig），
37KD，M

original

merged

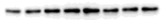

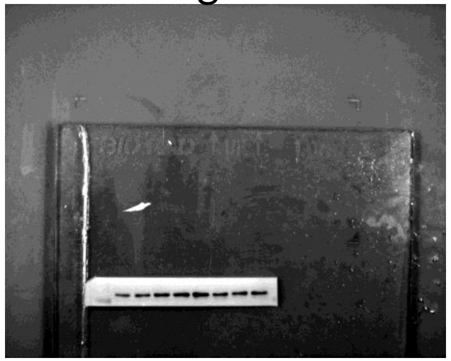

GAPDH（6004-1-Ig），
37KD，M

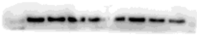

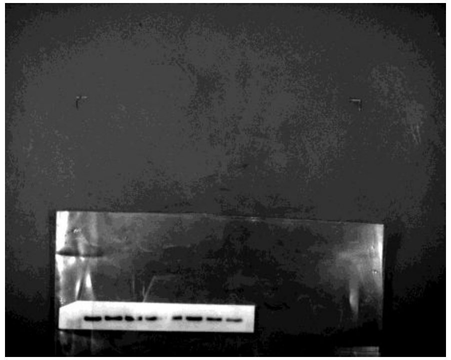

a-Tubulin (AF0001),
R, 55KD

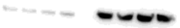

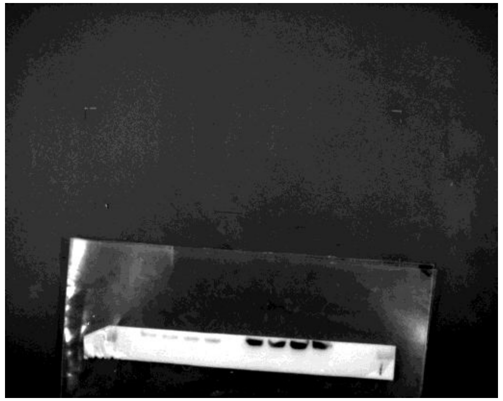

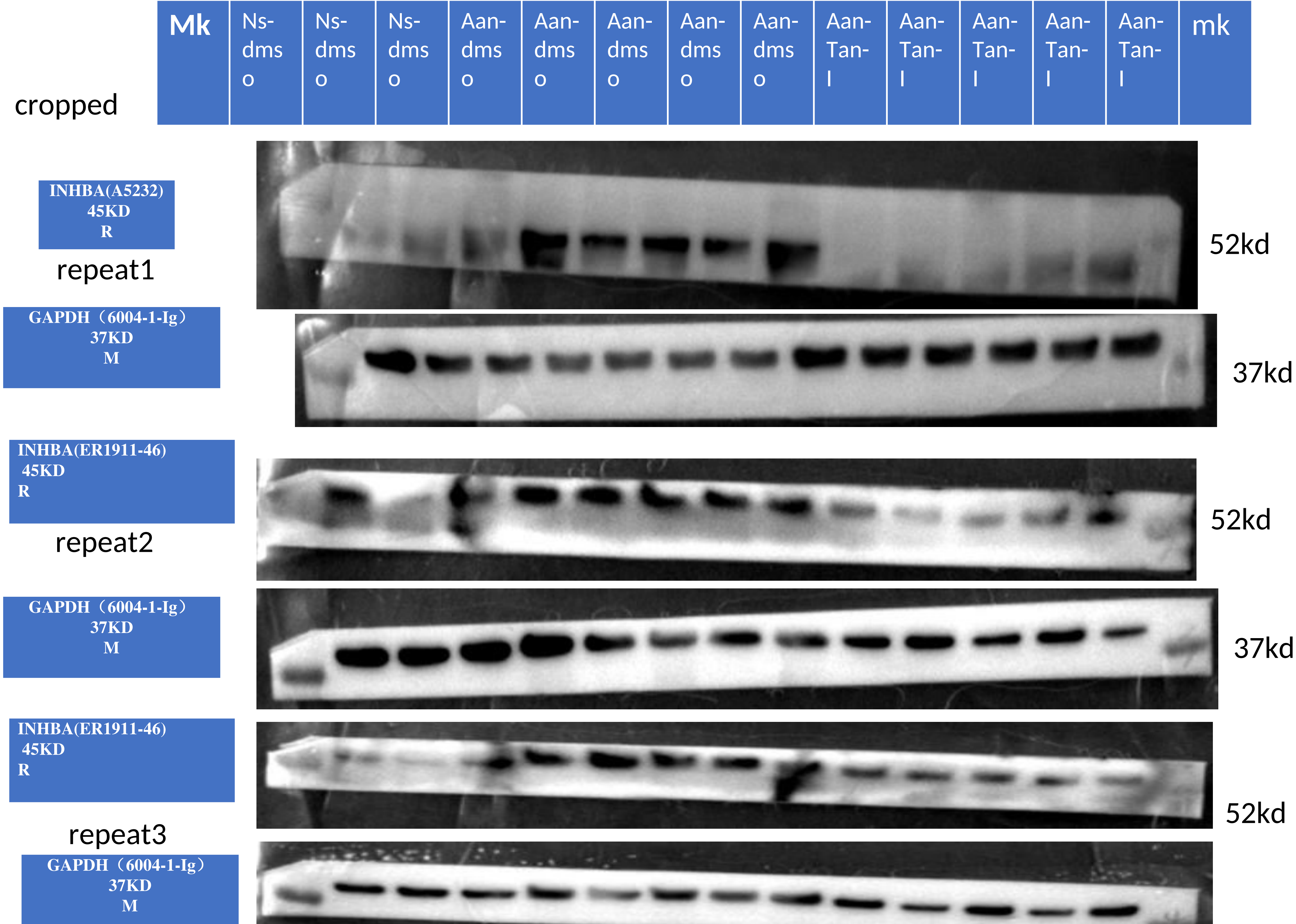

The expression of INHBA in NS or AAN kidneys was analyzed by Western blotting and then quantified.

original

merged

GAPDH（6004-1-Ig），
37KD，M

original

merged

INHBA(A5232)
45KD
R

repeat1

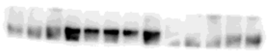

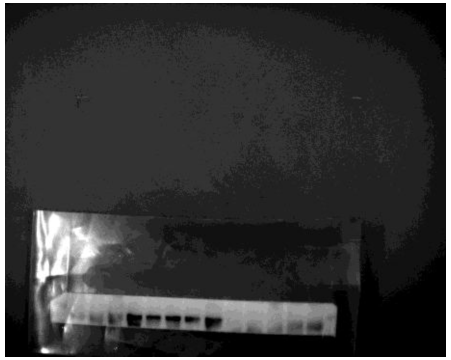

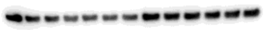

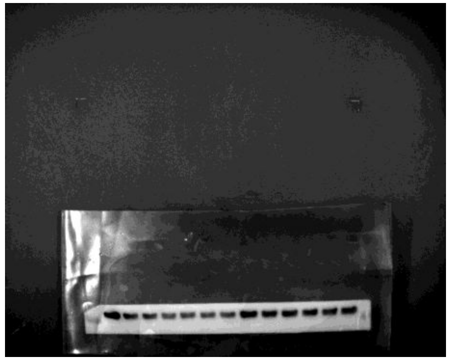

INHBA(A5232)
45KD
R

repeat2

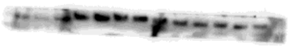

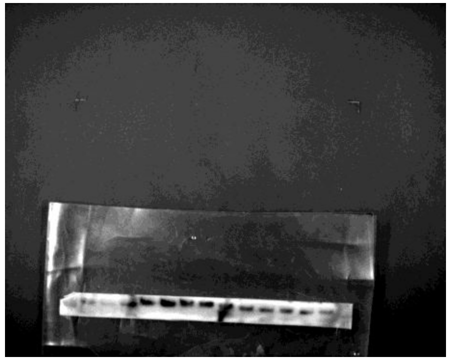

GAPDH（6004-1-Ig），
37KD，M

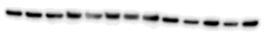

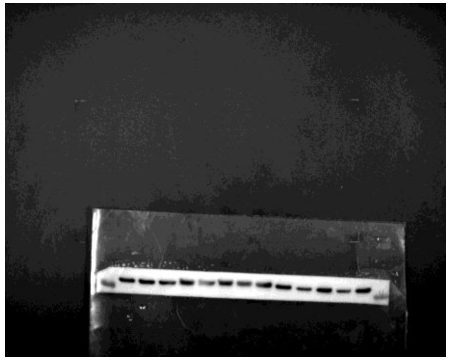

INHBA(A5232)
45KD
R

repeat3

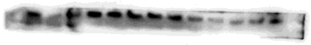

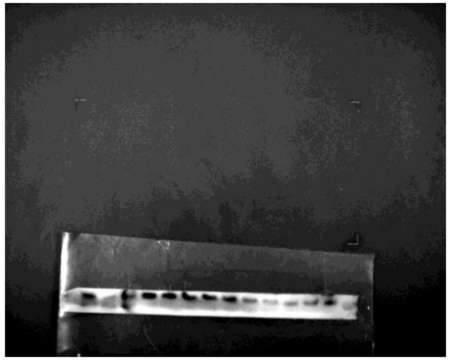

GAPDH（6004-1-Ig），
37KD，M

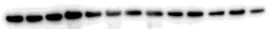

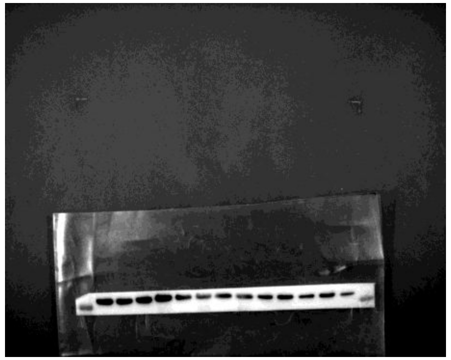

Supplement: Supplementary file 4 — Additional file 4. The original images of Western blot assay in figure 4 [file 12906_2022_3592_MOESM4_ESM.pdf]
