# Supplementary material for: Tanshinone I attenuates fibrosis in fibrotic kidneys through down-regulation of inhibin beta-A
Source: BMC Complement Med Ther. 2022 Apr 19;22:110. doi: 10.1186/s12906-022-03592-3 (PMC9020026; doi:10.1186/s12906-022-03592-3)

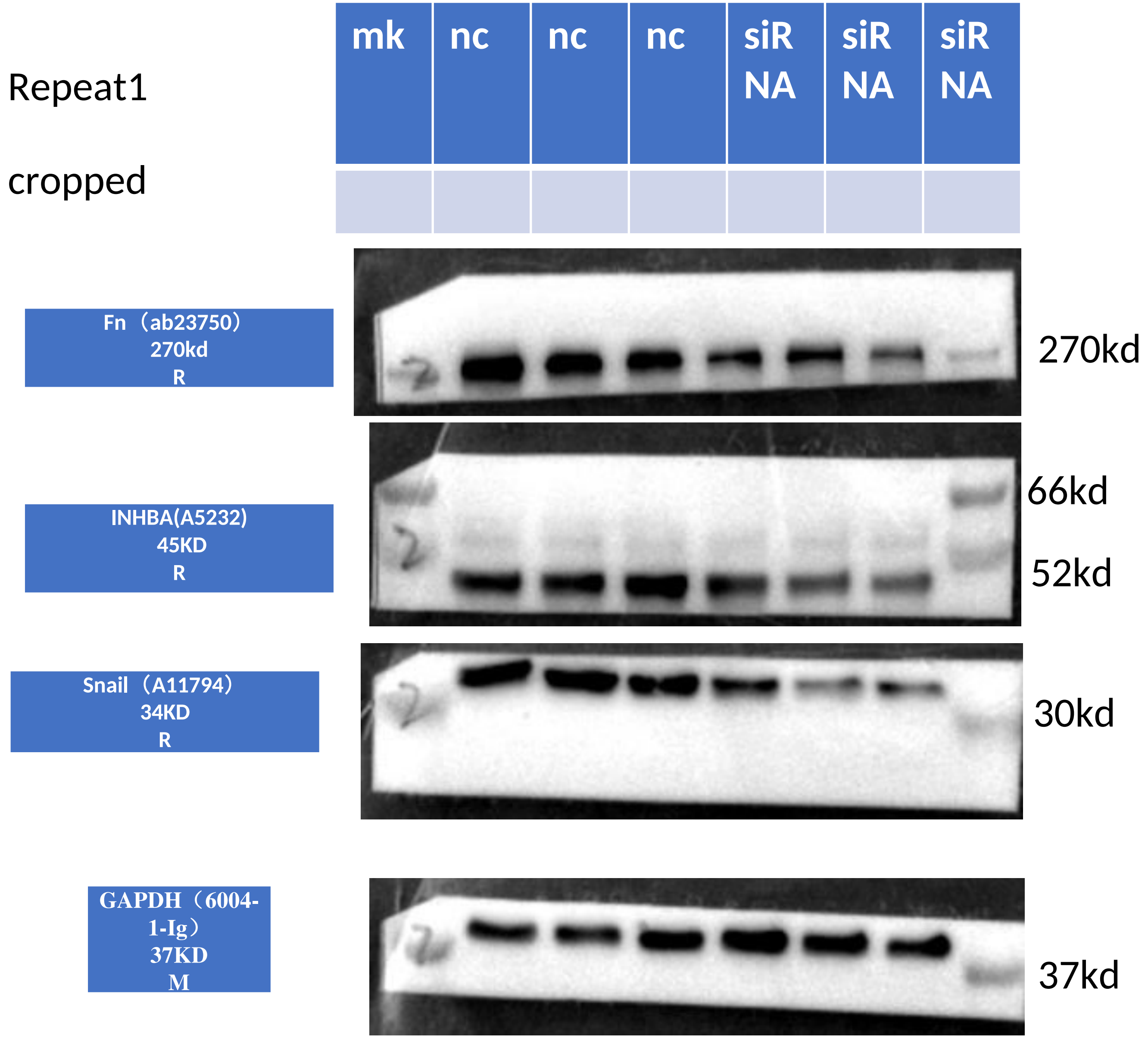

NRK-49F cells were transfected with nonsense control (NC) or INHBA siRNA (siINHBA). After 24h, cell lysates were collected and Western blot analysis was performed to measure the expression of INHBA, FN and Snail.

Fn（ab23750）
270kd
R

original

merged

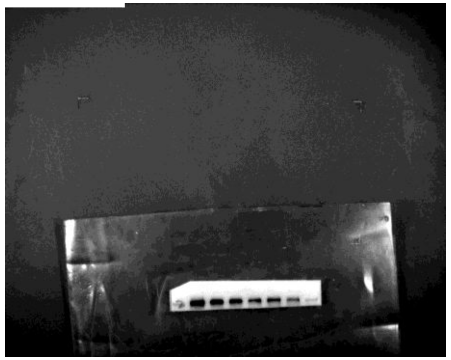

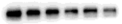

INHBA(A5232)
45KD
R

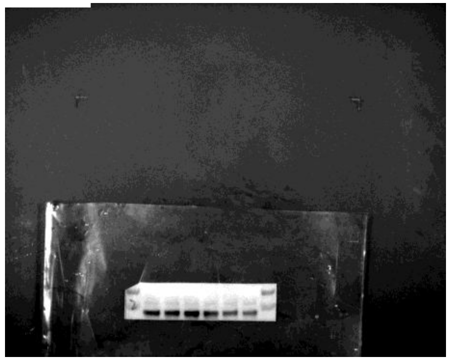

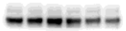

Snail（A11794）
34KD
R

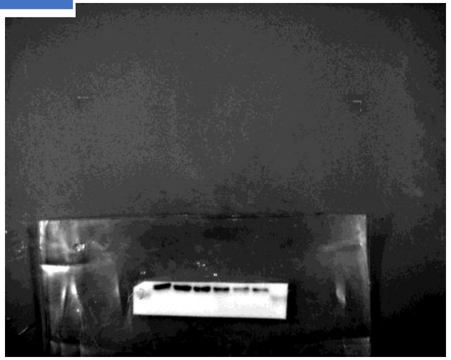

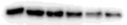

GAPDH（6004-1-Ig）
37KD
M

original

merged

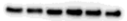

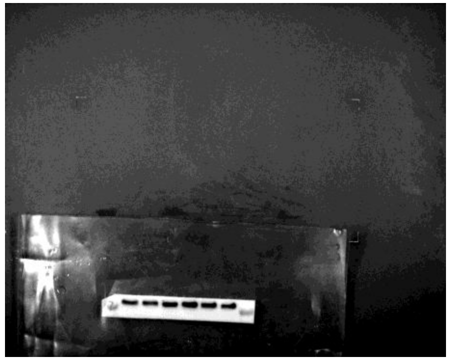

Repeat2

cropped

mk	Nc	Nc	nc	siR NA	siR NA	siR NA	mk

Fn（ab23750）
270kd
R

INHBA(A5232)
45KD
R

Snail（A11794）
34KD
R

GAPDH（6004-1-Ig）
37KD
M

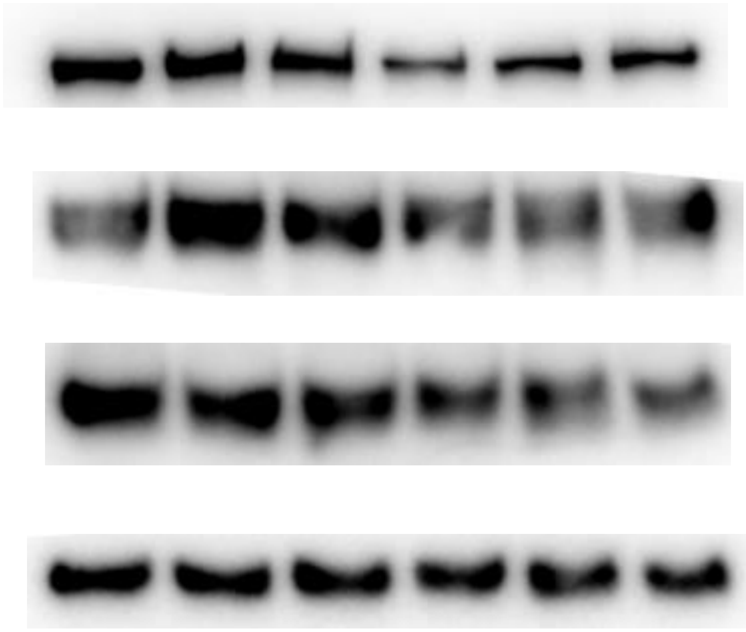

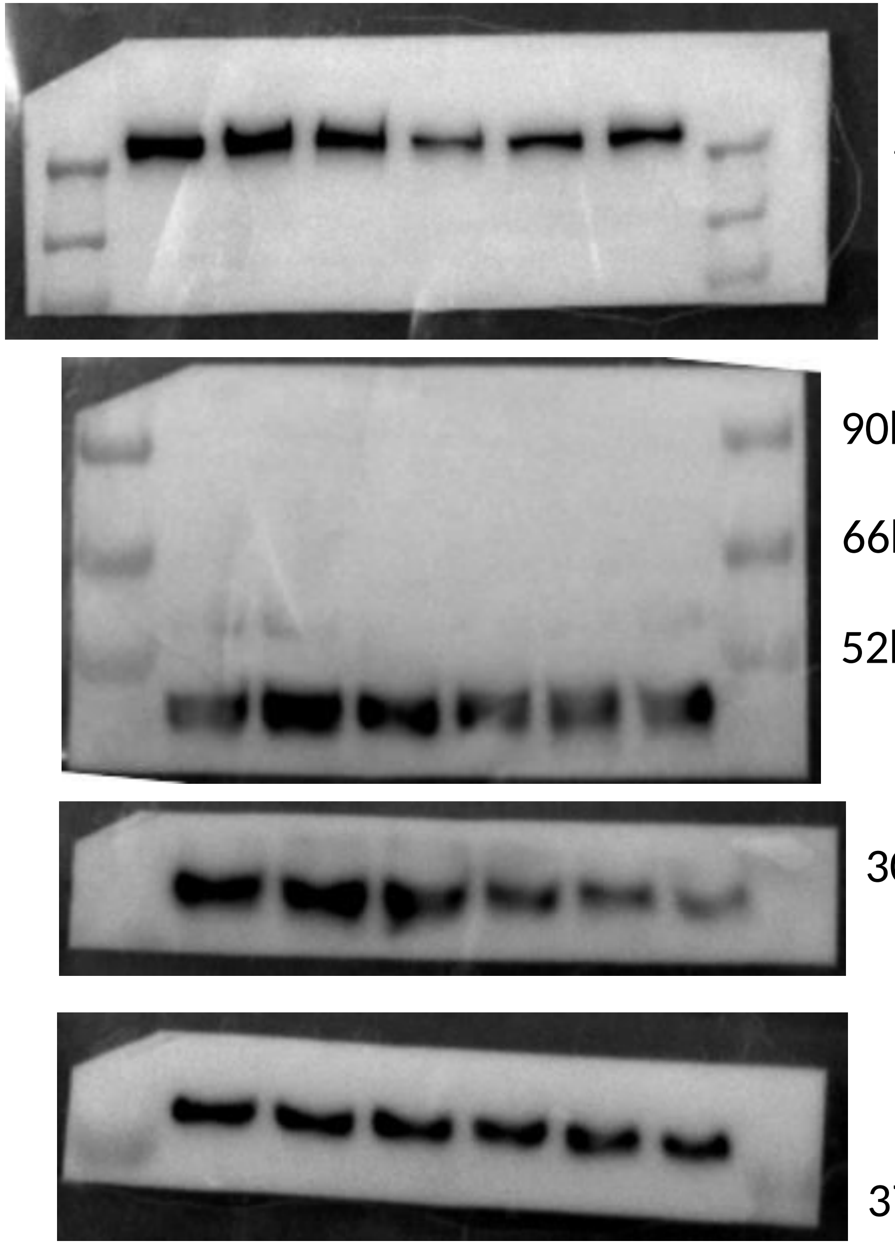

270kd
170kd
130kd
90kd
66kd
52kd
30kd
37kd

Fn（ab23750）
270kd
R

original

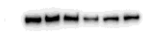

merged

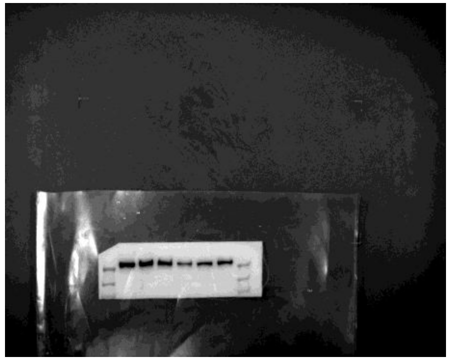

GAPDH（6004-1-Ig）
37KD
M

original

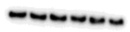

merged

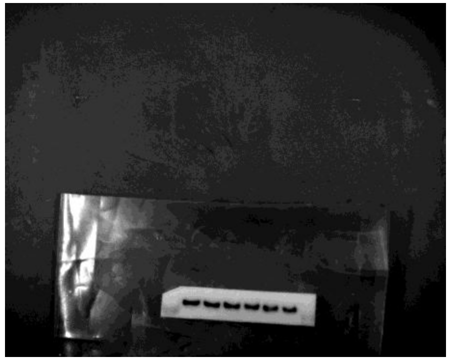

INHBA(A5232)
45KD
R

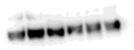

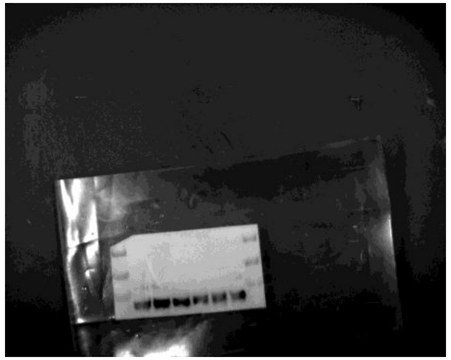

Snail（A11794）
34KD
R

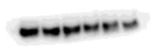

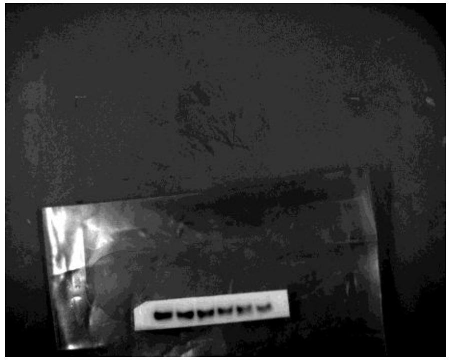

Repeat3

cropped

mk	Nc	Nc	siRNA	siRNA

Fn（ab23750）
270kd
R

INHBA(A5232)
45KD
R

Snail（A11794）
34KD
R

GAPDH（6004-1-Ig）
37KD
M

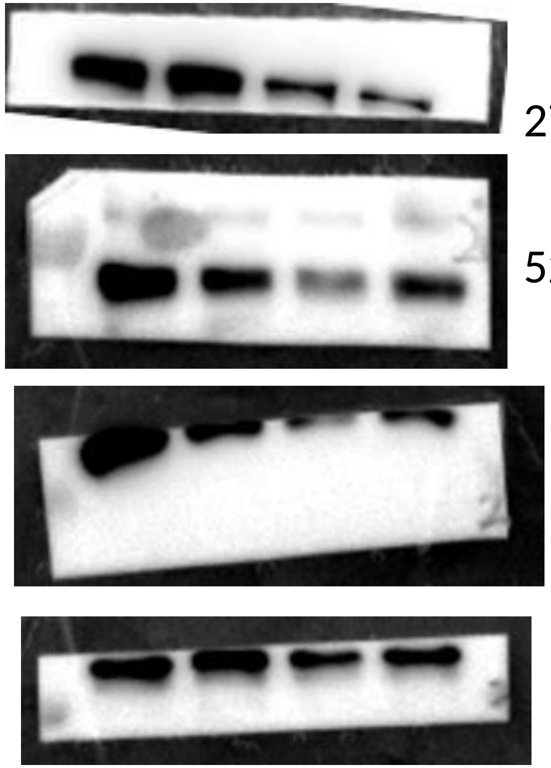

270kd

52kd

30kd

37kd

INHBA(A5232)
45KD
R

original

merged

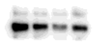

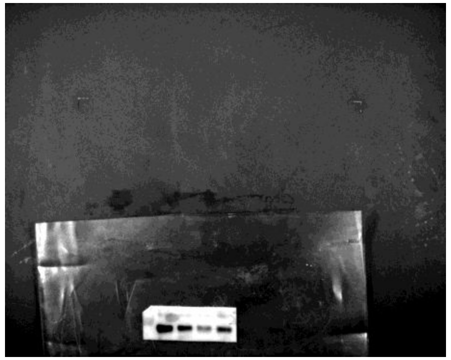

Snail（A11794）
34KD
R

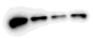

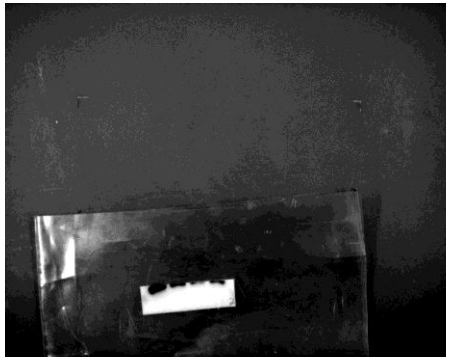

GAPDH（6004-1-Ig）
37KD
M

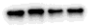

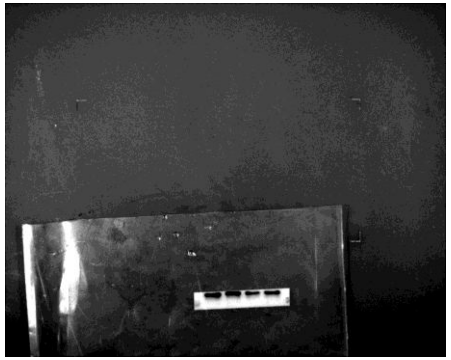

Fn（ab23750）
270kd
R

original

merged

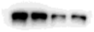

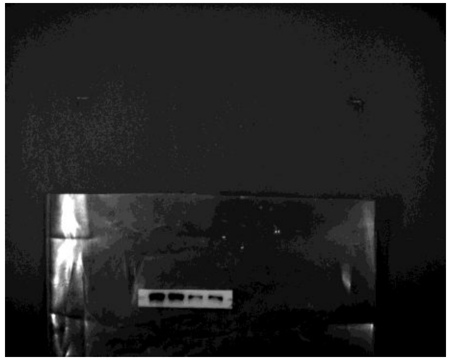

Repeat1

cropped

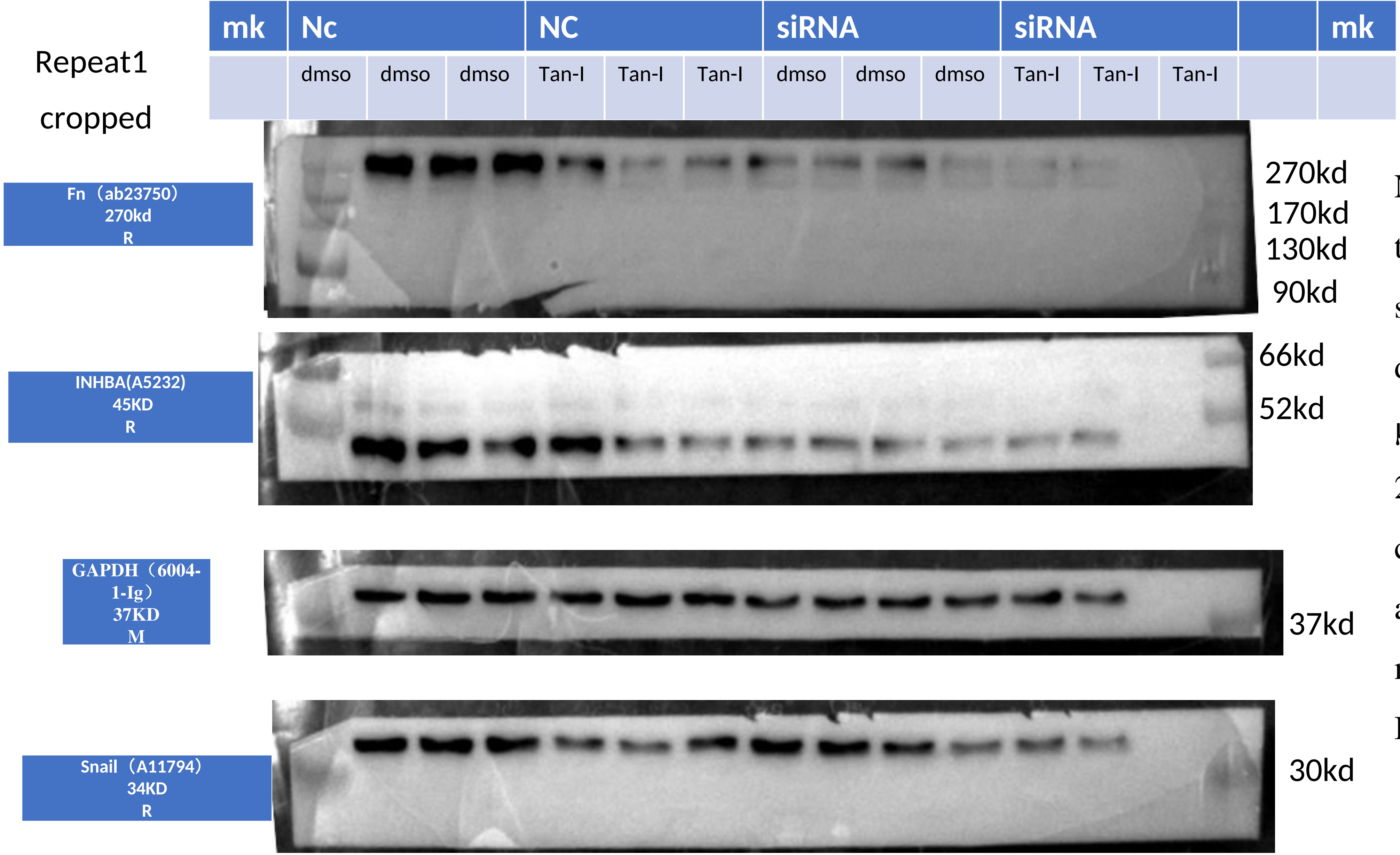

NRF-49F cells were transfected with NC or siINHBA. On the second day, cells were treated 50 μM of Tan-I for another 24h. Cell lysates were collected and Western blot analysis was performed to measure the expression of INHBA, FN and Snail.

Fn（ab23750）
270kd
R

original

merged

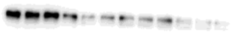

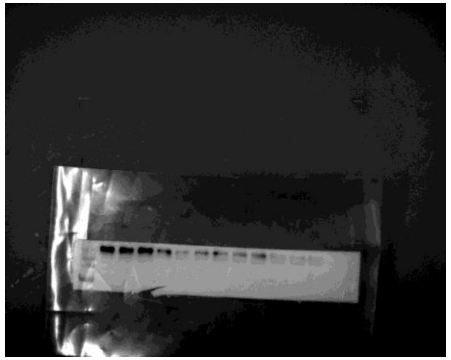

INHBA(A5232)
45KD
R

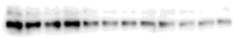

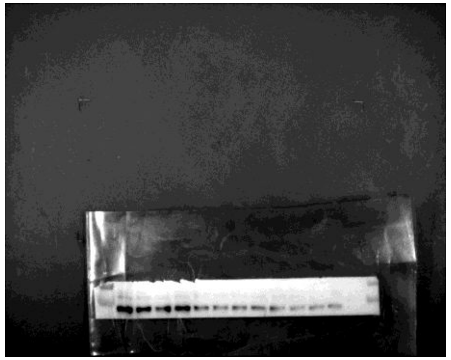

Snail（A11794）
34KD
R

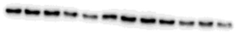

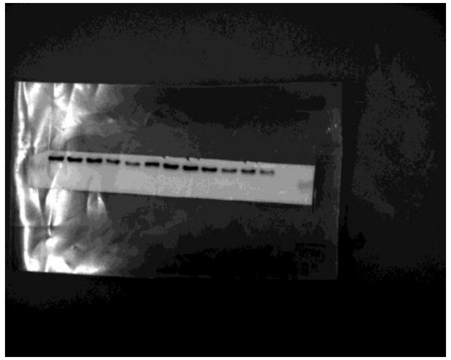

GAPDH（6004-1-Ig）
37KD
M

original

merged

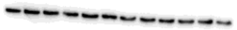

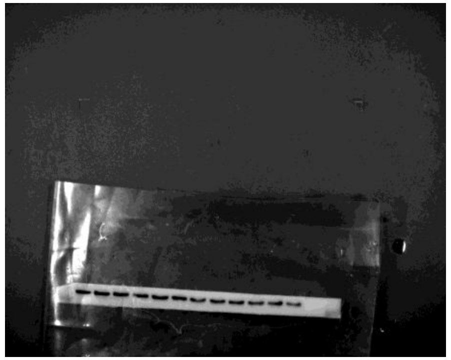

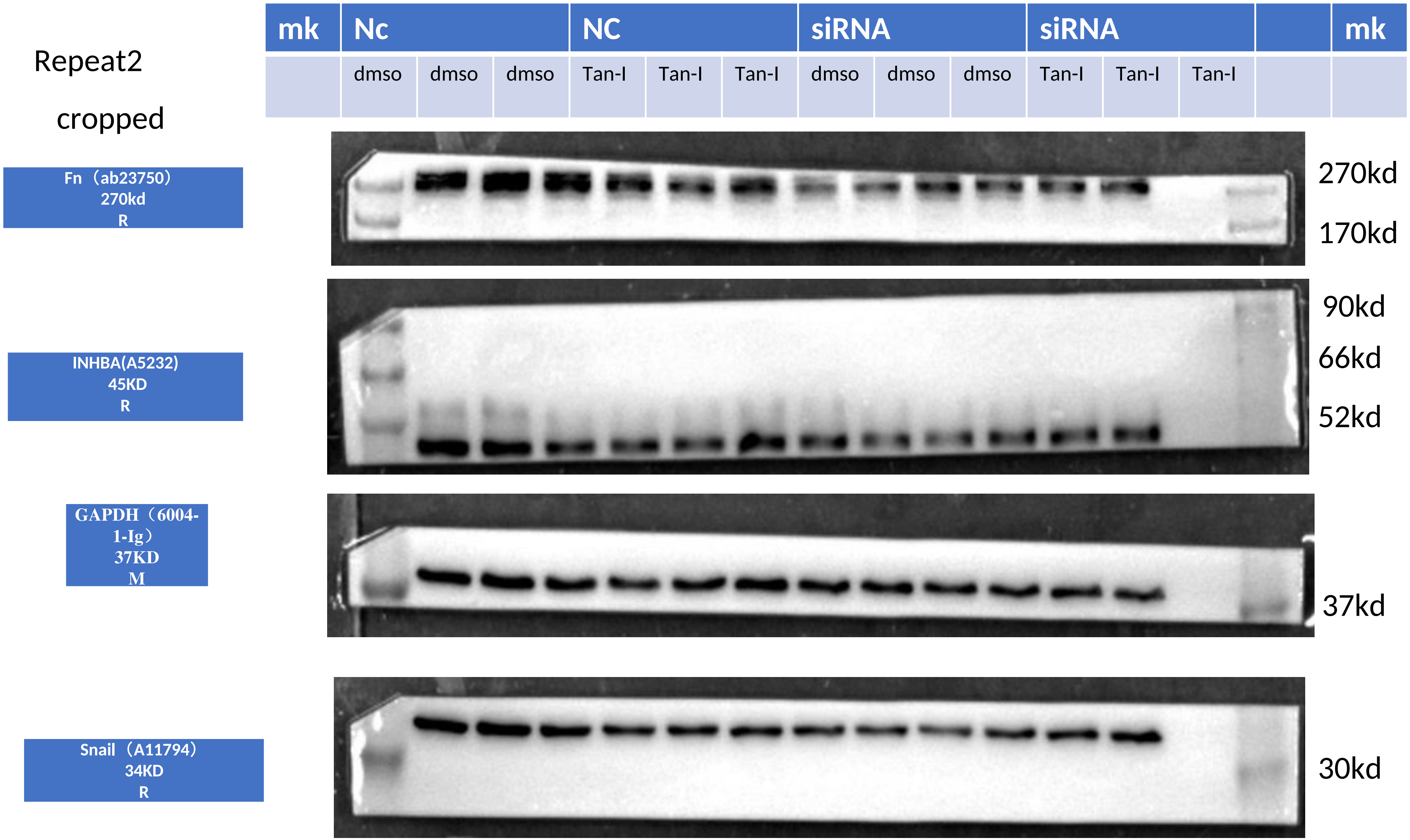
Repeat2
cropped
mk
Nc
NC
siRNA
siRNA
mk
dmso
dmso
dmso
Tan-I
Tan-I
Tan-I
dmso
dmso
dmso
Tan-I
Tan-I
Tan-I
Fn（ab23750）
270kd
R
270kd
170kd
INHBA(A5232)
45KD
R
90kd
66kd
52kd
GAPDH（6004-1-Ig）
37KD
M
37kd
Snail（A11794）
34KD
R
30kd

Fn（ab23750）
270kd
R

original

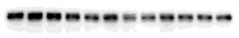

merged

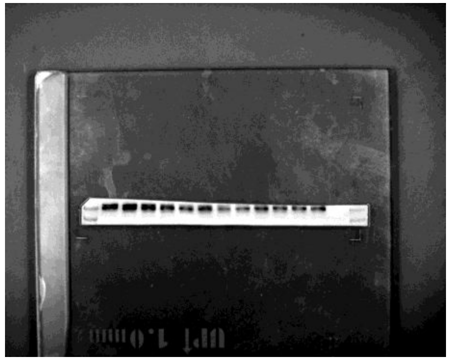

Snail（A11794）
34KD
R

original

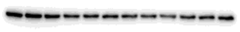

merged

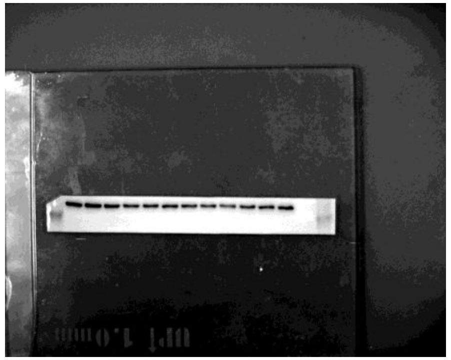

INHBA(A5232)
45KD
R

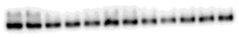

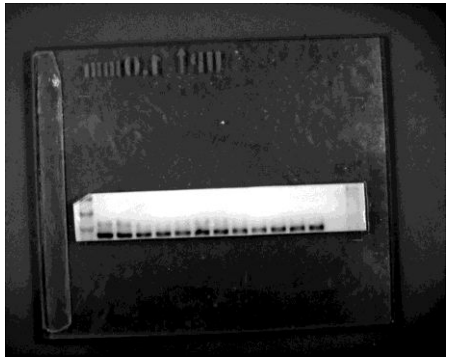

GAPDH（6004-1-Ig）
37KD
M

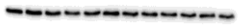

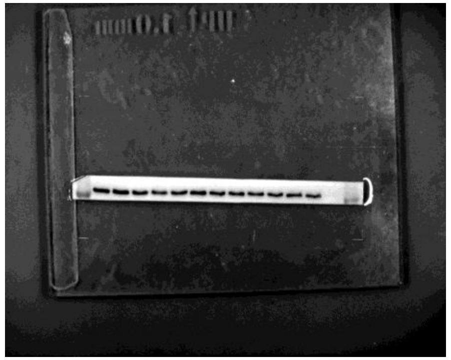

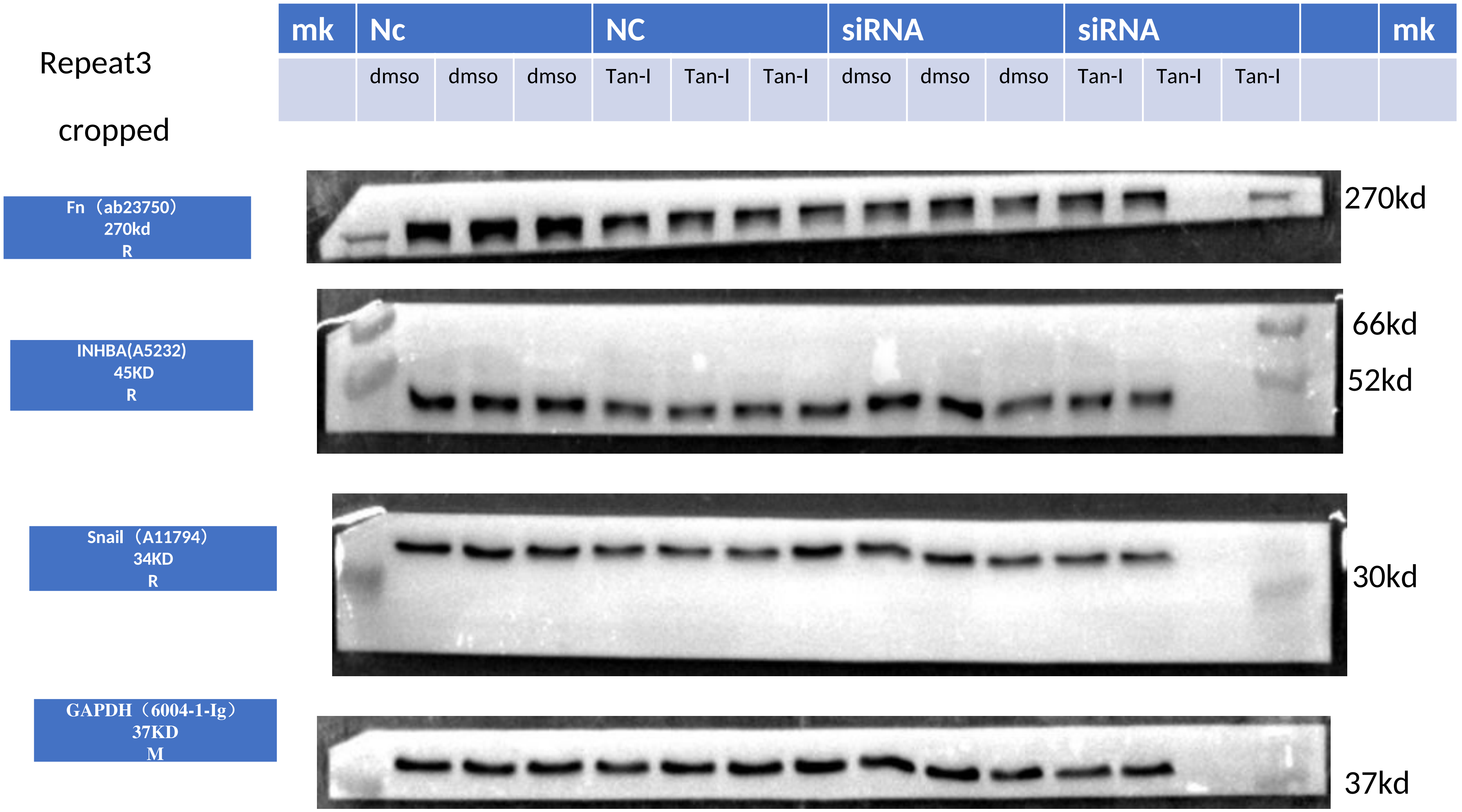
Repeat3
cropped
mk
Nc
NC
siRNA
siRNA
mk
dmso dmso dmso Tan-I Tan-I Tan-I dmso dmso dmso Tan-I Tan-I Tan-I
Fn（ab23750）
270kd
R
270kd
INHBA(A5232)
45KD
R
66kd
52kd
Snail（A11794）
34KD
R
30kd
GAPDH（6004-1-Ig）
37KD
M
37kd

Fn（ab23750）
270kd
R

original

merged

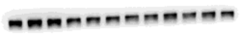

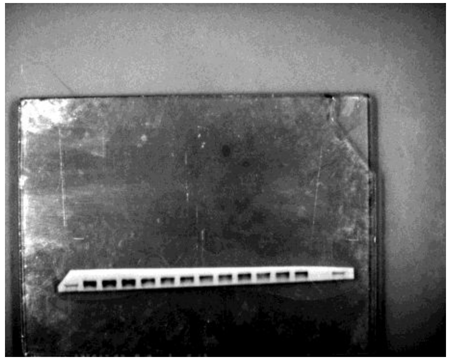

INHBA(A5232)
45KD
R

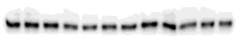

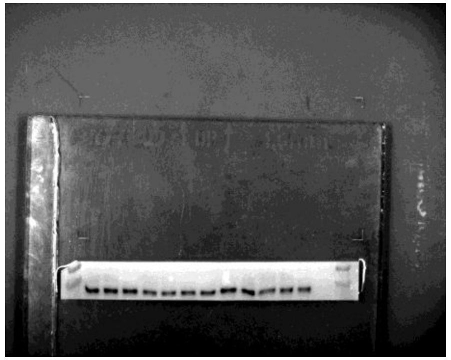

Snail（A11794）
34KD
R

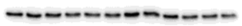

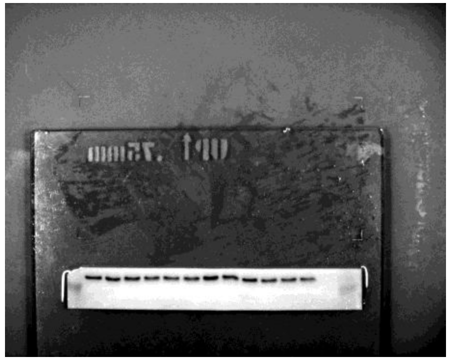

GAPDH（6004-1-Ig）
37KD
M

original

merged

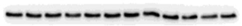

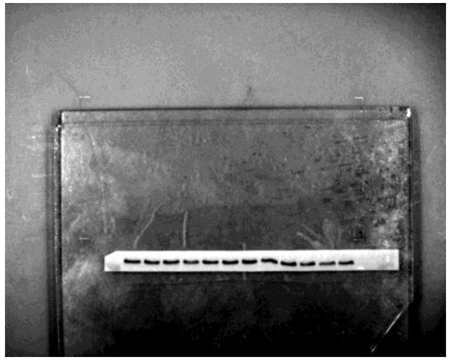

Supplement: Supplementary file 5 — Additional file 5. The original images of Western blot assay in figure 5 [file 12906_2022_3592_MOESM5_ESM.pdf]
